# Mesenchymal stem cells derived from human iPS cells via mesoderm and neuroepithelium have different features and therapeutic potentials

**DOI:** 10.1371/journal.pone.0200790

**Published:** 2018-07-25

**Authors:** Shinya Eto, Mizuki Goto, Minami Soga, Yumi Kaneko, Yusuke Uehara, Hiroshi Mizuta, Takumi Era

**Affiliations:** 1 Department of Cell Modulation, Institute of Molecular Embryology and Genetics, Kumamoto University, Kumamoto, Japan; 2 Department of Dermatology, Faculty of Medicine, Oita University, Yufu, Japan; 3 Department of Orthopedic Surgery, Graduate School of Medical Sciences, Kumamoto University, Kumamoto, Japan; University of Kansas Medical Center, UNITED STATES

## Abstract

Mesenchymal stem cells (MSCs) isolated from adult human tissues are capable of proliferating in vitro and maintaining their multipotency, making them attractive cell sources for regenerative medicine. However, the availability and capability of self-renewal under current preparation regimes are limited. Induced pluripotent stem cells (iPSCs) now offer an alternative, similar cell source to MSCs. Herein, we established new methods for differentiating hiPSCs into MSCs via mesoderm-like and neuroepithelium-like cells. Both derived MSC populations exhibited self-renewal and multipotency, as well as therapeutic potential in mouse models of skin wounds, pressure ulcers, and osteoarthritis. Interestingly, the therapeutic effects differ between the two types of MSCs in the disease models, suggesting that the therapeutic effect depends on the cell origin. Our results provide valuable basic insights for the clinical application of such cells.

## Introduction

Mesenchymal stem cells (MSCs) derived from embryonic mesoderm and neuroepithelium can be cultured in vitro to maintain their multipotency or be differentiated into three principle lineages: adipocyte, chondrocyte, and osteocyte [1–3]. In human and mouse adults, MSCs can be isolated from bone marrow, adipose tissue, and several other sites such as vascular pericytes [4].

MSCs isolated from adult tissues are valuable cell source for regenerative medicine because of their multipotency [5]. In addition, MSCs are used clinically in patients with graft-versus-host disease and various inflammatory conditions such as Crohn’s disease because of their modulatory effect on the immune response [6]. Indeed, clinical trials thus far have tested the efficacy of treatments with human MSCs for acute kidney failure, liver fibrosis, tendinitis, juvenile diabetes, radiation syndrome and rheumatoid arthritis, and inflammatory bowel disease [7,8].

Despite the progress in laboratory and clinical investigations, three major obstacles remain for the use of MSCs in patients. First, the procedures for harvesting MSCs from bone marrow or adipose tissues are sometimes invasive and can be dangerous for the patients [9]. Second, allogenic transplantations of MSCs designed to induce the patient’s immunological response are often rejected. Third, MSCs decrease in proliferative capacity and differentiation potential during long-term in vitro culture [10]. Therefore, novel culturing methods or alternative cell sources are needed to generate sufficient and safe MSCs for clinical use.

Human pluripotent stem cells (hPSCs), such as human embryonic stem cells (hESCs) and human induced pluripotent stem cells (hiPSCs), are potentially ideal sources for generating MSCs. HiPSCs and hESCs are similar in terms of morphology, self-renewal properties, and differentiation capacity; however, while hiPSCs can be easily and safely generated from adult host cells [11], the application of ESCs has faced substantial ethical and safety hurdles, culminating in their restricted use for clinical medicine [12]. Moreover, hiPSC-derived MSCs have several advantages compared to human tissue-derived MSCs for regenerative medicine. For example, human-derived MSCs are often limited in quantity, with average frequencies in the host population of 0.01–0.001% (bone marrow-derived MSCs; BM-MSCs) up to 1% (adipose tissue-derived MSCs; AT-MSCs) [13]. In contrast, hiPSCs can be easily generated in sufficient quantities from lymphocytes and skin-derived fibroblasts, and it is easier to preserve their high differentiation potential [11, 14]. Another advantage of hiPSCs over human tissue-derived MSCs is their high potential for therapeutic effect regardless of the donor’s age, while BM-MSCs or AT-MSCs from old donors can show diminished clonogenicity and lower differentiation capacity compared to MSCs derived from young donors [15, 16]. This difference lies in the rejuvenation that iPSCs undergo when they are reprogrammed to attain pluripotency [17], thus donor age in not such a factor for hiPSC-derived MSCs in term of differentiation capacity.

Several studies have been dedicated to establishing methods for inducing hiPSCs to MSCs [18, 19], although no reports have been published on the therapeutic potential of the MSCs derived from various origins in different disease models. We therefore sought to establish the methods for generating MSCs from hiPSC-derived mesoderm-like and neuroepithelium-like cells, and to evaluate their therapeutic effects in mouse disease models. This study also characterized MSCs grown from hiPSC-derived mesoderm-like and neuroepithelium-like cells.

## Materials and methods

### iPS cell culture

Human iPSCs, N1-12 [20] and 201B7 (RBRC-HPS0063 from RIKEN BRC BANK), were cultured with mitomycin C-treated mouse embryonic fibroblast feeder cells in human iPSC medium containing Dulbecco's modified Eagle’s medium: nutrient mixture F-12 (DMEM/F12) (SIGMA) supplemented with 20% KnockOut serum replacement (KSR, Invitrogen), 0.1 mM 2-mercaptoethanol (2-ME) (SIGMA), 2 mM L-glutamine (Life Technologies), 0.1 mM nonessential amino acids (NEAA, SIGMA), 0.5% penicillin and streptomycin (Nacalai Tesque, Japan), and 5 ng/ml basic fibroblast growth factor (bFGF, WAKO, Japan) [20]. Cells were harvested by treatment with CTK [1 mg/ml collagenase type IV (Invitrogen), 0.25% trypsin (Difco) and KSR] and passaged.

### Mesodermal and neuroepithelial differentiation from human iPSCs

For mesodermal differentiation, the semi-confluent human iPSCs were treated with CTK and transferred to petri dishes to form embryoid bodies (EB) in DMEM/F12 supplemented with 20% KSR, 2 mM L-glutamine, 0.1 mM NEAA, 0.1 mM 2-ME, 10 ng/ml bone morphogenetic protein 4 (BMP4) (R&D), and 0.5% penicillin and streptomycin. After 24 hours, the EB cells were spread in type IV collagen-coated dishes (Corning) and the medium was replaced with alpha modification of Eagle's medium **(**αMEM) (Gibco) supplemented with 10% FBS, 5 ng/ml bFGF, 0.1 mM 2-ME, 10 ng/ml BMP4, 3 ng/ml Activin A (GFH6-100), 5 mM LiCl (R&D), and 0.5% penicillin and streptomycin. Medium change was performed on day 2 and day 4. On day 6, the differentiated cells were treated with TrypLE express (Gibco) and reacted with antibodies against PDGFR-α-biotin antibody (Biolegend) and VEGFR2-PE antibody (R&D). For analysis and PSP purification, the cells were run through a FACS Aria III (BD Pahrmingen). The purified PSP population was resuspended in type IV collagen-coated dishes in αMEM supplemented with 10% FBS and 0.1 mM 2-ME.

For neuroepithelial differentiation, the semi-confluent human iPSCs were also treated with CTK and transferred to petri dishes for EB formation in DMEM/F12 supplemented with 20% KSR, 2 mM L-glutamine, 0.1 mM NEAA, 0.1 mM 2-ME, 10 ng/ml BMP4, and 0.5% penicillin and streptomycin. After 24 hours, the EB cells were distributed on type IV collagen-coated dishes and the medium was replaced with αMEM supplemented with 10% FBS and 0.1 mM 2-ME. 10^−7^ M RA (Invitrogen) was added daily from day 5 to day 8, and the medium was changed on days 3, 5, 6, and 8. On day 10, the PDGER-α+ and VEGFR2- fractions were purified by FACS Aria III, and then resuspended in type IV collagen-coated dishes in αMEM supplemented with 10% FBS and 0.1 mM 2-ME.

### Osteogenic differentiation

For differentiation into osteocytes, MSCs derived from hiPSCs were seeded at the density of 50,000 cells/cm^2^ in 24-well plates containing 0.5 ml of DMEM supplemented with 10% FBS, 10 mM β-glycerophosphate (SIGMA), 100 nM dexamethasone (SIGMA), 50 μM ascorbic acid (SIGMA), and 10 ng/ml BMP4, as described previously [2,21]. The plates were kept in a humidified incubator at 37°C and 5% CO_2_, and the culture medium was changed twice a week. After the 28-day period, the medium was removed and the cells were washed with PBS. After fixation of the cells with 4% paraformaldehyde (PFA) for 10 min, the cells were washed three times with distilled water, and then stained with 0.1% Alizarin red S for 10 min at room temperature (RT).

### Chondrogenic differentiation

For differentiation into chondrocytes, MSCs derived from iPSCs were seeded at 50,000 cells/cm^2^ in 24-well plates containing 0.5 ml of αMEM supplemented with 10% FBS, 0.1 mM 2-ME, 170 μM ascorbic acid, 100 nM dexamethasone, 10 ng/ml tumor growth factor beta 3 (TGF-β3) (R&D), and 10 ng/ml BMP2 (R&D). The plates were kept in a humidified incubator at 37°C and 5% CO_2_ as described previously [2,21], and the culture medium was changed twice a week. After the 28-day period, the medium was removed and the cells were washed with PBS. After fixation of the cells with 4% PFA for 10 min, the cells were washed three times with distilled water, and then stained with 0.5% alcian blue, containing 0.1 M HCl for 24 hours at RT.

### Adipogenic differentiation

For differentiation into adipocytes, MSCs derived from iPSCs were seeded at 20,000 cells/cm^2^ in 12-well plates containing 1 ml of DMEM supplemented with 10% FBS, 1 mM dexamethasone, 5 mg/ml insulin (SIGMA), 500 mM 3-isobutyl-1-methylxanthine (IBMX) (SIGMA), and 10 mM indomethacin (SIGMA), as described previously [2,21]. The plates were kept in a humidified incubator at 37°C and 5% CO_2_, and the culture medium was changed twice a week. After fixation of the cells with 4% PFA for 10 min, the cells were washed three times with distilled water, and then stained with Oil Red O for 10 min at RT.

### CFU-F assay

PSP-MSCs and RA-Pα-MSCs (300 cells of each) were seeded in type IV collagen-coated 6-well plates containing 1 ml of αMEM supplemented with 10%FBS and 0.1 mM 2-ME, as described previously [2,22]. After 2 weeks, the colonies were stained with Leishman’s eosin methylene blue solution modified (Merck KGaA) for colony counting.

### Flow cytometry

MSC marker expression in the PSP-MSCs and RA-Pα-MSCs was investigated by flow cytometry using the FACS Aria III, following incubation for 30 min at 4°C with the following monoclonal antibodies: CD34-FITC, CD45-APC, CD49d-PE, CD73-PE, CD90-APC, CD106-APC (BD Pharmingen), CD105-biotin, CD140α-biotin, and CD140β-biotin (Biolegend), CD271 (BD pharmingen), anti-VIMENTIN and anti-STRO1 (Abcam). Streptavidin APC (Bioscience) was used as the secondary antibody for the biotin-labeled primary antibodies.

### RNA isolation and PCR analysis

Total RNAs was purified by Sepasol^®^ Super G reagent (Nacalai Tesque, Japan). Total RNA was transcribed to DNA with Superscript III (Invitrogen) and random primers (Invitrogen). Quantitative polymerase chain reaction (qPCR) was performed with THUNDERBIRD^TM^ qPCR Mix (TOYOBO), and the data were analyzed with the StepOnePlus real-time PCR system (Applied Biosystems). The list of primers is supplied in S1 Table.

### Skin wound and pressure ulcer model

To generate the skin wound model and pressure ulcer model, we used immunodeficient mice, 6-week-old NOG (In-Vivo Science Inc.), and 8-week-old NOJ mice (Kyudo company) [23], respectively. For the skin wound model, dorsal surfaces of the mice were shaved and a 0.8-cm diameter skin biopsy tool (Vernier Calipers) was used to create a full-thickness excision wound that extended to the fascia, before subcutaneously injecting 1.0×10^6^ hiPSC-derived MSCs around each wound. Photographs were taken from day 0 to day 14 and the wound healing was measured using the area of ellipse formula (0.5 × length of Major axis) (0.5 × length of Minor axis) (π) [24]. Wound bearing animals were housed individually during the course of the experiment [25]. For pressure wound model, the dorsal skin of immunodeficient mice was pulled up and placed between two cylinders of magnets (Seiko Sangyo Co., Ichikawa, Japan), producing a compressive pressure of 50 mmHg between the magnets [26]. After skin treatment by 16-hour ischemia and subsequent reperfusion, the skin ulcer develops until day 5. As shown in previous case, we subcutaneously injected our MSCs around the ulcer. Photographs were taken from day 5 to day 20 and measurement were carried out as for the skin wound model. All mice were maintained under standard conditions at Center for Animal Resources and Development (CARD) at Kumamoto University. The mice received food and water ad libitum and were maintained on a 12/12-hour light/dark cycle. Two weeks after the MSCs transplantation, the mice from both models were sacrificed and the skin wounds were isolated for analysis. Skin tissues were embedded in O.C.T compound (Sakura finetek USA). All mice used in this experiments were euthanized by cervical dislocation.

### Osteoarthritis model

To generate the osteoarthritis model in 8-week-old immunodeficient (NOJ) male mice, the anterior cruciate ligament (ACL) was transected and medial meniscus was removed from the joints of the lower limbs [27, 28]. Two weeks later after the operation, we injected 1.0 × 10^5^ MSCs dissolved with hyaluronic acid into the disrupted joints. Two weeks later, the mice were sacrificed and the knee tissues were isolated for analysis by fixation in 4% PFA followed by safranin O staining. The damage was calculated using a modified Mankin score [29]. All mice used in these experiments were euthanized by cervical dislocation.

### Hematoxylin and eosin staining and immunostaining of ulcer model tissues

After fixation with 4% PFA, the different ulcer wound tissues were sectioned longitudinally at 10-μm thickness, and then either stained with hematoxylin and eosin (HE) or immunostained using the human MHC class I antibody (ab52922; Abcam) at 1:50 dilution, followed by anti-rabbit Alexa flora 488 (Invitrogen) secondary antibody at 1:3,000 dilution and then nuclear staining with 1 ng/ml Hoechst 33258 (Invitrogen).

### Microarray analysis

We isolated 200 ng of total RNA from the N1-12 iPSCs, BM-MSCs (PRC-010; Bay bioscience), PSP-MSC and RA-Pα-MSC derived from N1-12 and 201B7 for biotin labeling and fragmentation according to the manufacturer’s protocol (3’ IVT Express kit, Affymetrix). Biotin labelling samples were hybridized to a GeneChip® Human Genome U133 Plus 2.0 (Affymetrix). Then, the arrays were scanned by a GeneChip® Scanner 3000 (Affymetrix), and the array data were analyzed with GeneSpring GX 12.5 software (Agilent technologies). Each data were normalized to the median of the measurements and the threshold value was set to 100. Using GeneSpring GX 12.5 software, the genes with a fold change > 2.0 were considered to be differentially expressed genes between the iPSC-derived MSCs (PSP-MSC and RA-Pα-MSC) and N1-12 iPSCs. Comparing the profiles of differentially expressed genes revealed up-regulated and down-regulated genes in both PSP-MSCs and RA-Pα-MSCs. GO categorization was applied based on the GeneSpring GX 12.5 software GO database terms of up-regulated and down-regulated genes, selected and described solely based on corrected *P*-value ranking. The datasets of all genes investigated were clustered according to Euclidean distance metrics, using the gene list imported by the Import Entity list supplied with the GeneSpring GX 12.5 software. The list of pluripotent markers, MSC markers, and paracrine factors are shown in S2 Table. The pluripotent markers included the characterization markers for iPSCs [30], upregulated genes in ESI mass spectrometry for iPSCs [31], and “Yamanaka factors” [32]. MSC markers contained characterization markers of MSCs for regenerative medicine [33–35] and comparative markers for MSCs among their original tissue [36]. The paracrine factors included immunoregulatory cytokines [37] and regenerative medicine-related factors [38, 39].

### Ethical approval

All experimental procedures involving human samples and animal experiments were approved by the following ethics committees at Kumamoto University: Human Genome and Gene Analysis Research in the Faculty of Life Sciences, Clinical Research and Advanced Medical Technology, and Epidemiological and General Research in the Faculty of Life Science.

### Teratoma formation

The PSP-MSCs and RA-Pα-MSCs, induced from iPSCs of N1-12 and 201B7, were harvested and dissolved in medium containing 10% collagenase. Next, we injected 1.0 x 10^6^ cells into the testis of NOJ mice [20], and 11 weeks later the teratoma samples were harvested and fixed in PBS containing 10% formalin for paraffin-embedding and HE staining.

### Immunofluorescence staining of MSC markers

For immunostaining, cells were fixed with PBS containing 4% PFA for 10 min at RT, washed two times with PBS, and then incubated overnight at 4°C in PBS containing 2% BSA with CD34-FITC, CD45-APC, CD49d-PE, CD73-PE, CD90-APC, CD106-APC, CD271, CD105-biotin, CD140α-biotin, CD140β-biotin, anti-VIMENTIN, and anti-STRO-1. In some cases, the cells were washed two times with PBS containing 2% BSA and incubated with Streptavidin APC or anti-mouse Alexa Flora 488 (Abcam). Finally, the cell nuclei were stained with 1 ng/ml Hoechst 33258 and the cells were images for data analysis with IN CELL ANALYZER 6000 (GE Healthcare). The antibody information is described in the **Flow cytometry** chapter.

## Results

### Generating MSCs from mesoderm-like cells using in vitro human iPSC culture

We previously identified and purified mesoderm-like cells in an in vitro mouse ESC culture, visualizing platelet-derived growth factor receptor α (PDGFRα) and vascular endothelial growth factor receptor 2 (VEGFR2) as surface markers [21]. Based on their gene expressions and fate analyses, PDGFRα+/VEGFR2- cells, named herein as PDGFRα single positive cell (PSP), and PDGFRα-/VEGFR2+ cells, named herein as VEGFR2 single positive cell (VSP), represented paraxial mesoderm- and lateral mesoderm-types of cells, respectively [21]. In mouse development, the paraxial mesoderm differentiates into mesenchyme descendants such as osteocytes, chondrocytes, and skeletal muscle cells that are also MSC progenies; from this, we proposed that hiPSC-derived PSP could give rise to MSCs.

**Fig 1:**
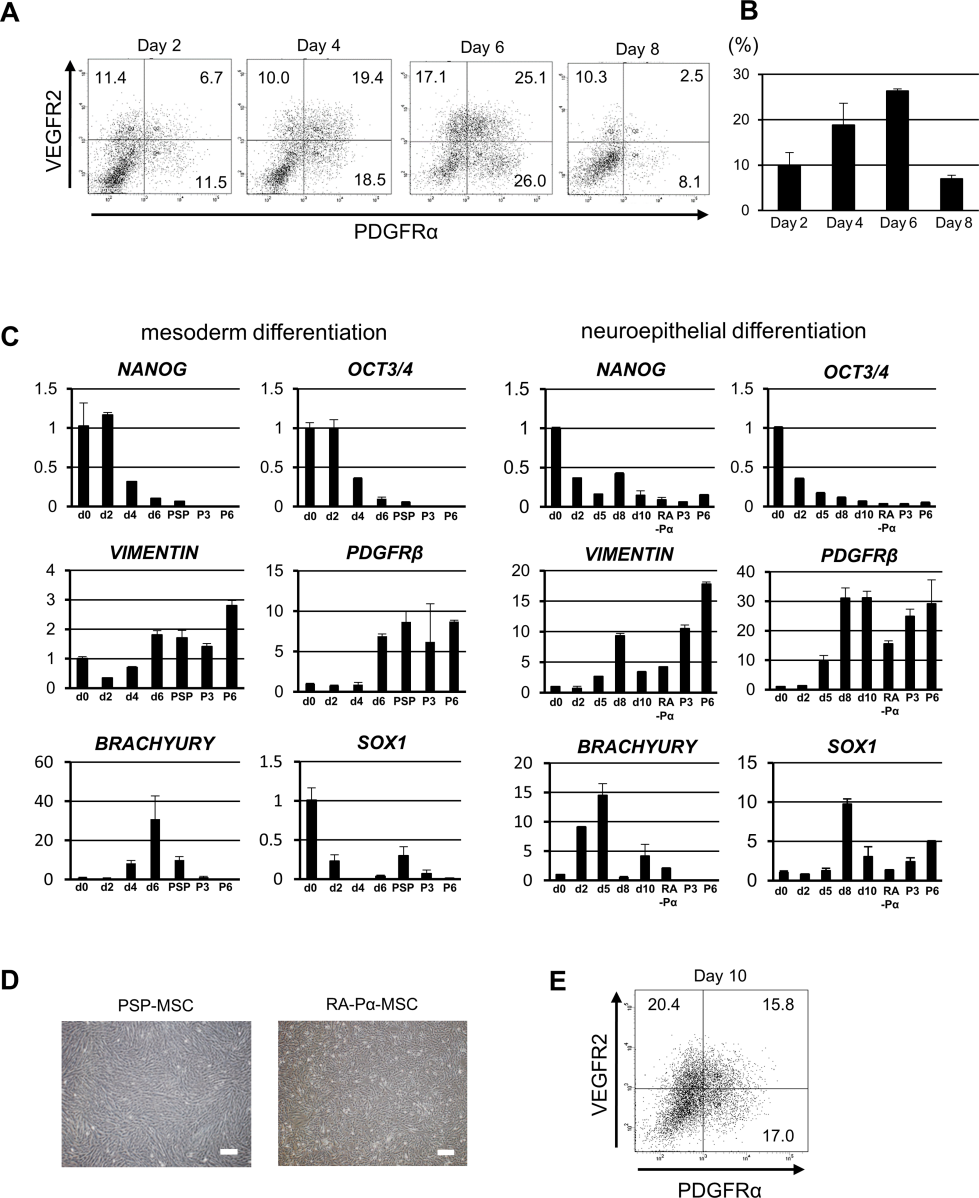
Induction of hiPSC-derived MSCs under mesodermal and neuroepithelial differentiation conditions. (A, B): The proportions of PDGFRα and VEGFR2 expression in differentiated N1-12 cells on day 2, 4, 6 and 8 under the mesodermal differentiation condition. Representative data (A) and graph (B). Number indicates the percentage of each population (A). Experiments were conducted three times (mean ± SD). (C): Quantitative PCR (qPCR) analysis of the relative mRNA levels of NANOG and OCT3/4 as multipotent markers, VIMENTIN and PDGFRβ for mesenchyme, BRACHYURY for mesoderm, and SOX1 for neuroepithelium during the mesodermal (left) and neuroepithelial (right) differentiations. Symbols: d-number indicates day-number of differentiation, PSP; immediately after sorting on day 6 under the mesoderm differentiation, RA-Pα: immediately after sorting on day 10 under the neuroepithelial differentiation, P3 or P6; passage 3 or 6 after sorting. Each value represents the mean fold compared to day-0 undifferentiated iPSCs. Each experiment was conducted three times (mean ± SD). (D): Bright-light image of PSP-MSC and RA-Pα cells on day 21 after sorting. Scale bar: 200 μm. (E): The proportion of PDGFRα and VEGFR2 expressions in day-10 differentiated N1-12 under the neuroepithelial differentiation condition; number indicates the percentage of each population.

To prove this hypothesis, we first attempted to induce PSP in an in vitro hiPSC culture. Healthy donor-derived hiPSCs, N1-12 and 201B7 [20, 32], formed the embryoid bodies (EBs) following treatment with bone morphogenetic protein 4 (BMP4) for 24 hours. EBs were then spread on collagen type IV-coated dishes and cultured in the presence of BMP4, basic fibroblast growth factor (bFGF), activin A, and LiCl for 5 days. During iPSC differentiation, expressions of mesoderm markers, PDGFRα and VEGFR2, gradually elevated to achieve peak levels on day 6, when we purified PSP (day 6-PSP) (Fig 1A and 1B and S1A Fig). While the expression levels of pluripotent markers, *NANOG* and *OCT3/4*, decreased gradually during the mesodermal differentiation, the mesodermal marker, *BRACHYURY*, increased up to day 6, and then decreased (Fig 1C).

After culturing the PSP cells for one month under the MSC differentiation, they exhibited a typical spindle-shape morphology resembling human dermal fibroblasts and sustained growth in vitro (Fig 1D). We monitored the marker expressions of day 6-PSP after purification by flow cytometry (Fig 1C). The mesenchymal markers, *PDGFRβ* and *VIMENTIN*, increased in expression as culturing proceeded, while *BRACHYURY* decreased and disappeared. In contrast, *SOX1*, a neuroepithelial marker, was not expressed during this mesodermal differentiation process of MSCs from iPSCs.

### Induction of hiPSC-derived MSC under neuroepithelial differentiation conditions

We previously demonstrated neuroepithelium as a site of origin for MSCs in mouse development and that Sox1+ neuroepithelium-like cells could give rise to MSCs in vitro in mouse ESC cultures [2]. Retinoic acid (RA) is a strong inducer for neuroepithelium-like cells during in vitro PSC differentiation. Herein, we first determined an appropriate time of RA treatment during in vitro hiPSC differentiation (S1B Fig). Treating with RA from day 5 to day 8 induced the highest expression levels of PDGFRα under neural differentiation conditions (Fig 1E, S1B and S1C Fig). Culturing the PDGFRα+/VEGFR2- (RA induced PDGFRα single positive cells; RA-Pα) cells after purification by flow cytometry, the RA-Pα cells easily altered their morphology to appear like human skin-derived fibroblasts (Fig 1D). *PDGFRβ* and *VIMENTIN* expressions were also much higher in these derived cells than in the PSP-derived cells (Fig 1C). In contrast to the mesodermal differentiation, *SOX1* expression was dramatically enhanced with RA treatment and increased gradually in RA-Pα cells as the culture proceeded (Fig 1C). Thus, we established two distinct types of mesenchymal cells using an in vitro hiPSC differentiation culture.

### The hiPSC-derived fibroblast-like cells fulfill the MSC criteria

**Fig 2:**
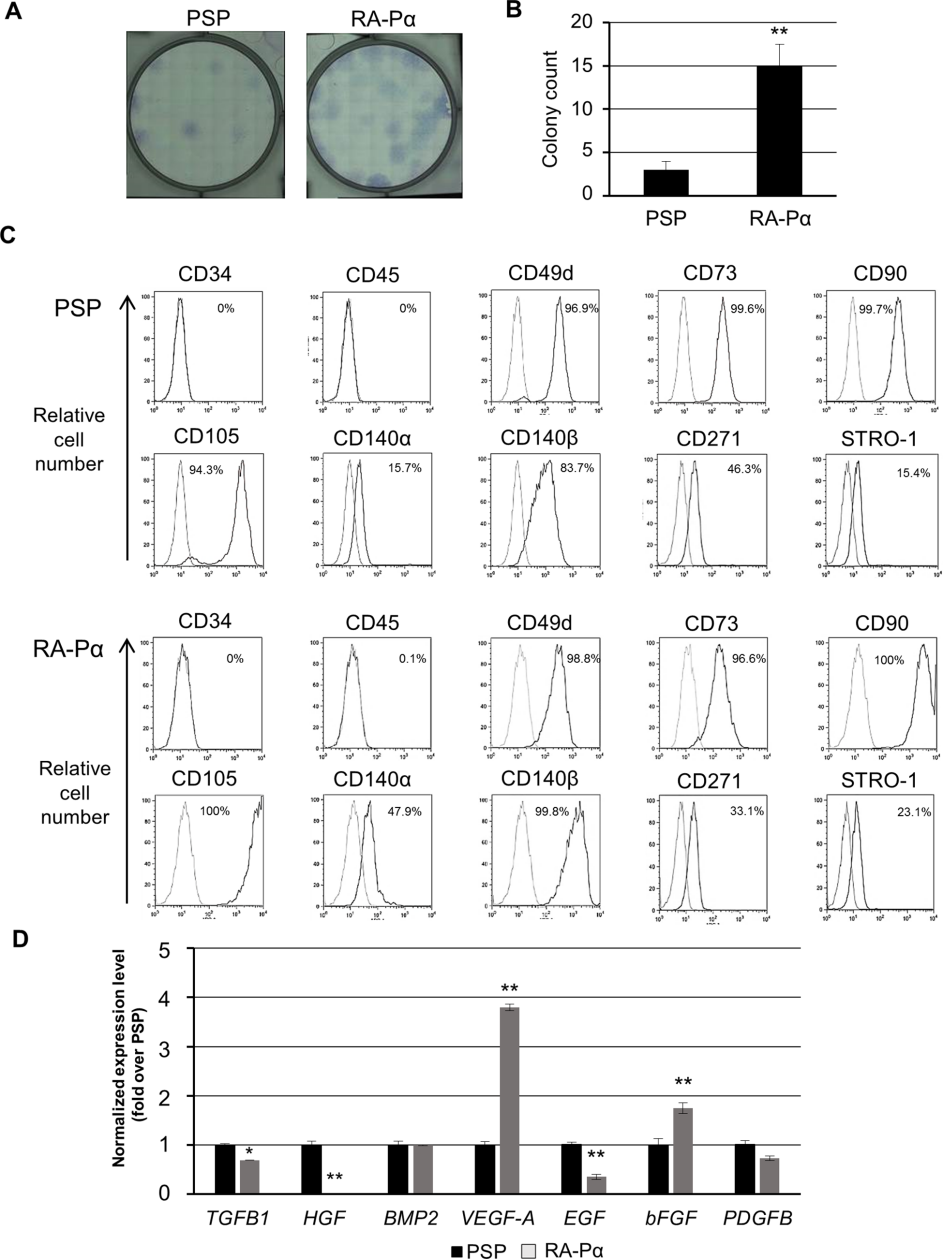
Characterization of PSP- and RA-Pα-derived mesenchymal stem cells. (A): Imaging of CFU-F formation in the PSP- and RA-Pα-derived MSCs. (B): CFU-F numbers for the PSP- and RA-Pα-derived MSCs. 300 cells were spread in each 6-well dish. Colony number was calculated on day 14 (n = 3, mean ± SD). **P < 0.01. (C): Analysis of marker expressions in PSP- and RA-Pα-derived MSCs by flow cytometer. MSC-related markers: CD49d, CD73, CD90, CD105, CD140α, CD140β, CD271 and STRO-1; hematopoietic markers: CD34 and CD45. (D): Analysis of paracrine factor expressions in PSP- and RA-Pα-derived MSCs by qPCR. TGFB1: tumor growth factor beta 1, HGF: hepatocyte growth factor, BMP2: bone morphogenetic protein 2, VEGF-A: vascular endothelial growth factor A, EGF: epidermal growth factor, bFGF: basic fibroblast growth factor, and PDGFB: platelet-derived growth factor beta. Data are means ± SDs of three independent experiments. *P < 0.05, **P < 0.01, compared between PSP- and RA-Pα-derived MSCs (t test).

We next examined the colony-forming potential of hiPSC-derived mesenchymal cells induced under both the conditions established in this study. MSCs can form fibroblastic colonies in vitro, known as colony forming units for fibroblast (CFU-F). Both of the derived mesenchymal cells formed CFU-Fs, although the unit number in RA-Pα cell-derived mesenchymal cells was significantly higher than that in PSP-derived mesenchymal cells (Fig 2A and 2B).

Flow cytometry and immunostaining analysis then revealed that both the PSP- and RA-Pα-derived mesenchymal cells expressed a set of markers specific for MSCs and mesenchymal cells (CD49d, CD73, CD90, CD105, CD140α, CD140β, CD271, STRO-1 and VIMENTIN), but not the vascular endothelial and hematopoietic markers, CD34 and CD45, respectively (Fig 2C and S2 Fig). According to recent studies, MSCs express some growth factors that can support their own growth, in a paracrine effect [39–41]. We thus investigated the expression of factors involved in MSC-paracrine growth in our hiPSC-derived mesenchymal cells. Hepatocyte growth factor (HGF) was more strongly expressed in PSP-derived mesenchymal cells compared to RA-Pα-derived mesenchymal cells, while vascular endothelial growth factor-A (VEGF-A) and bFGF were more strongly expressed in RA-Pα-derived mesenchymal cells compared to PSP-derived mesenchymal cells (Fig 2D).

**Fig 3:**
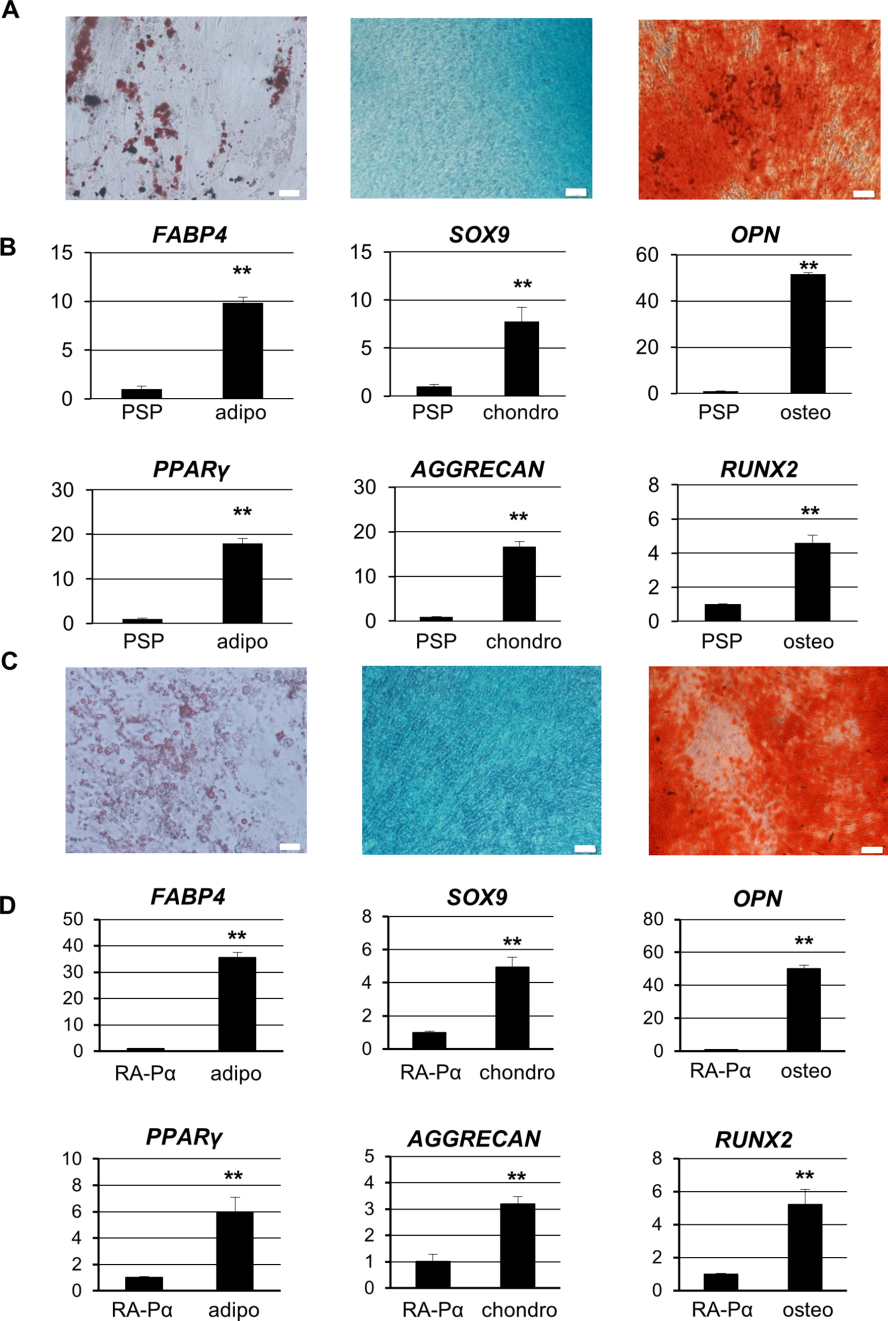
Differentiation potential of PSP- and RA-Pα-derived MSCs in vitro. (A-D): Differentiation of PSP- (A and B) and RA-Pα-derived (C and D) MSCs into adipocytes, chondrocytes, and osteocytes. Representative staining of adipocytes (Oil red O staining, left panels in A and C), chondrocytes (alcian blue staining, middle panels in A and C) and osteocytes (Alizarin red staining, right panels in A and C). Scale bar = 40 μm, Relative expression levels of lineage-specific markers by qPCR (B and D); FABP4 and PPARγ for adipocyte, SOX9 and AGGRECAN for chondrocyte, and OPN and RUNX2 for osteocyte. Each experiment was conducted in triplicate (mean ± SD). **P < 0.01, compared with undifferentiated PSP-MSCs (B) or RA-Pα-MSCs (D) (t test). Symbols: PSP; undifferentiated PSP-MSCs, RA-Pα; undifferentiated RA-Pα-MSCs, adipo; day 28 under adipocyte differentiation, chondro; day 28 under chondrocyte differentiation, osteo; day 28 under osteocyte differentiation.

We then accessed the potential for differentiation into adipocyte, chondrocyte, and osteocyte lineages in both types of hiPSC-derived mesenchymal cells cultured under the appropriate respective conditions. The cultured cells were positive for the specific staining and highly expressed the specific markers for adipocytes, chondrocytes, and osteocytes, respectively (Fig 3A–3D). These results suggested that our hiPSC-derived mesenchymal cells have the potential to differentiate into adipocytes, chondrocytes, and osteocytes. Of note, our MSCs did not form tumors when transplanted into the testes of immunodeficient mice, suggesting no malignant potential (S3A–S3C Fig). Taken together, these findings indicated that both hiPSC-derived mesenchymal cells established herein fulfill the MSC criteria.

### Characterization of hiPSC-derived MSCs

**Fig 4:**
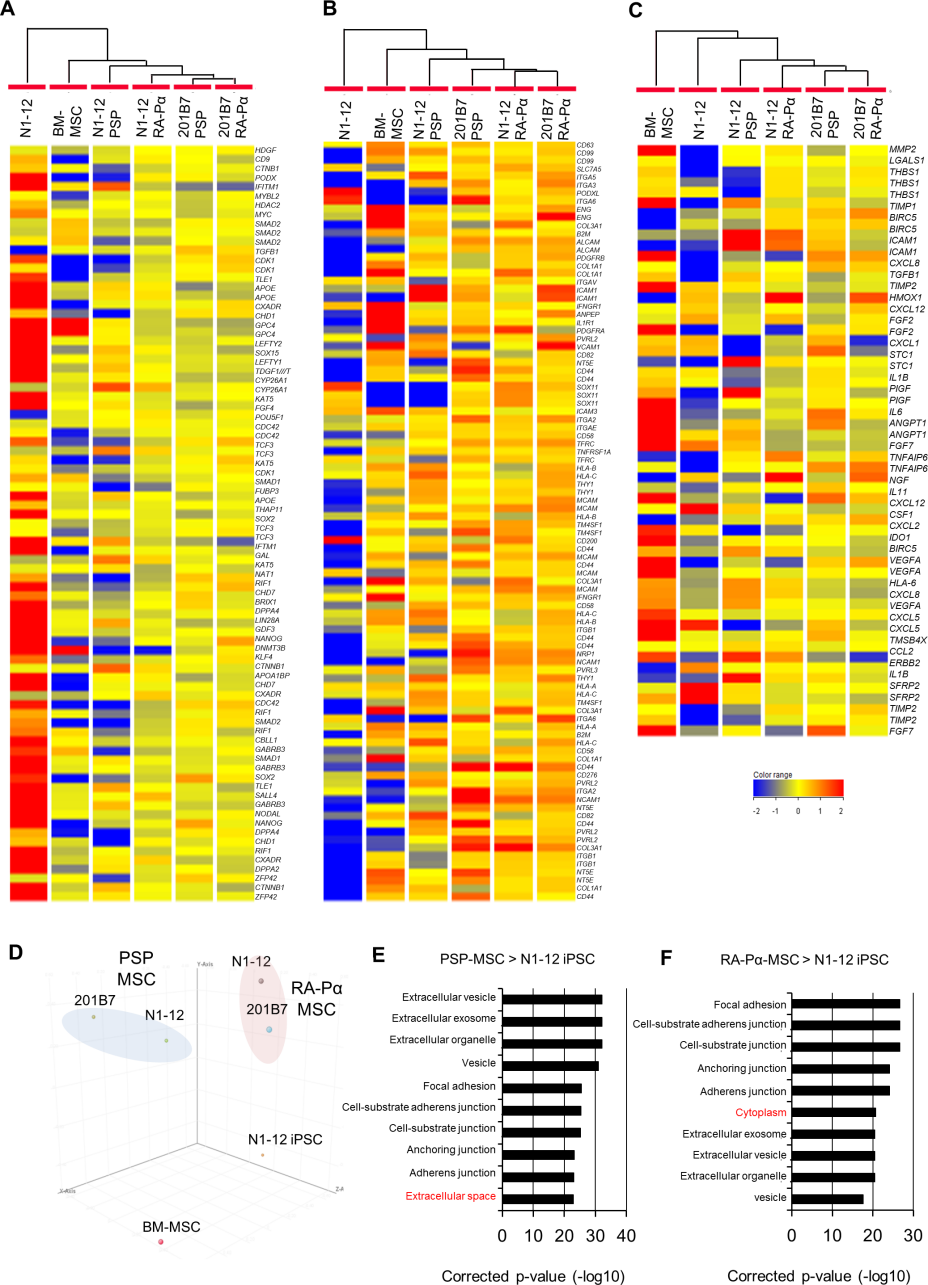
DNA microarray analysis of PSP-MSCs and RA-Pα-MSCs. (A-C): Hierarchical clustering of gene sets signatures, pluripotent markers (A), MSC markers (B) and paracrine factors (C), in various MSCs and N1-12 iPSCs. The datasets of all genes investigated were clustered according to Euclidean distance metrics. The labels represent the following cells: N1-12; N1-12 iPS cells, BM-MSC; BM-MSC (PRC-010) purchased from Bay bioscience Co.; N1-12 PSP; PSP-MSC derived from N1-12, 201B7 PSP; PSP-MSC derived from 201B7, N1-12 RA-Pα; RA-Pα-MSC derived from N1-12, 201B7 RA-Pα; RA-Pa-MSC derived from 201B7. (D): Principal component analysis. All datasets were classified into three principal components, PC2 (25.43%), PC3 (15.34%), and PC4 (7.81%), and were simplified into three-dimensional scores. (E, F): Gene ontology (GO) analysis of 286 commonly upregulated data sets for PSP-MSC (E) and 359 data sets for RA-Pα-MSC (F). The top-ten GO terms are listed. GO terms were detected with a cutoff P-value of 0.1. Values are–log10 corrected P-value. Red color indicates different GO terms between (E) and (F).

To further characterize our PSP-MSCs and RA-Pα-MSCs, we compared their genome-wide expression patterns with those of human iPSCs and BM-MSCs using DNA microarrays. We selected the iPSC, MSC markers, and paracrine factors according to previous studies [30–39] (Fig 4A–4C). Hierarchical expression clustering revealed relatively higher expression levels of MSC markers in the PSP-MSCs and RA-Pα-MSCs, and lower expression levels of iPSC markers, compared to the iPSCs (Fig 4A and 4B). We confirmed that N1-12 iPSC expressed a set of pluripotent markers at a similar level to the 201B7 iPSC (S4A Fig). These results suggest that our methods succeeded in inducing MSCs from human iPSCs. The expression patterns of MSC markers are classified based on their differentiation procedures (Fig 4B). In contrast, the expression patterns of paracrine factors of MSCs are classified based on their iPSC origin (Fig 4C). Principal component analysis (PCA) confirmed that both PSP-MSCs and RA-Pα-MSCs are closer to each other than to the other cell types, suggesting that the similarity in global gene expression is more dependent on the origin of MSC than the parental iPSCs (Fig 4D). We also searched for the up- and downregulated genes of PSP-MSCs and RA-Pα-MSCs compared with iPSCs (S4B–S4E Fig), and gene ontology (GO) analysis revealed that many GO terms overlapped between the PSP-MSCs and RA-Pα-MSCs (Fig 4E and 4F, S4F and S4G Fig). Taken together, these expression results suggested that some gene signature patterns in PSP-MSCs are similar to those in RA-Pα-MSC, while the global expression pattern of the derived MSCs is more dependent on the cells of origin.

### Effect of hiPSC-derived MSCs in an *in vivo* mouse model of skin ulcer

**Fig 5:**
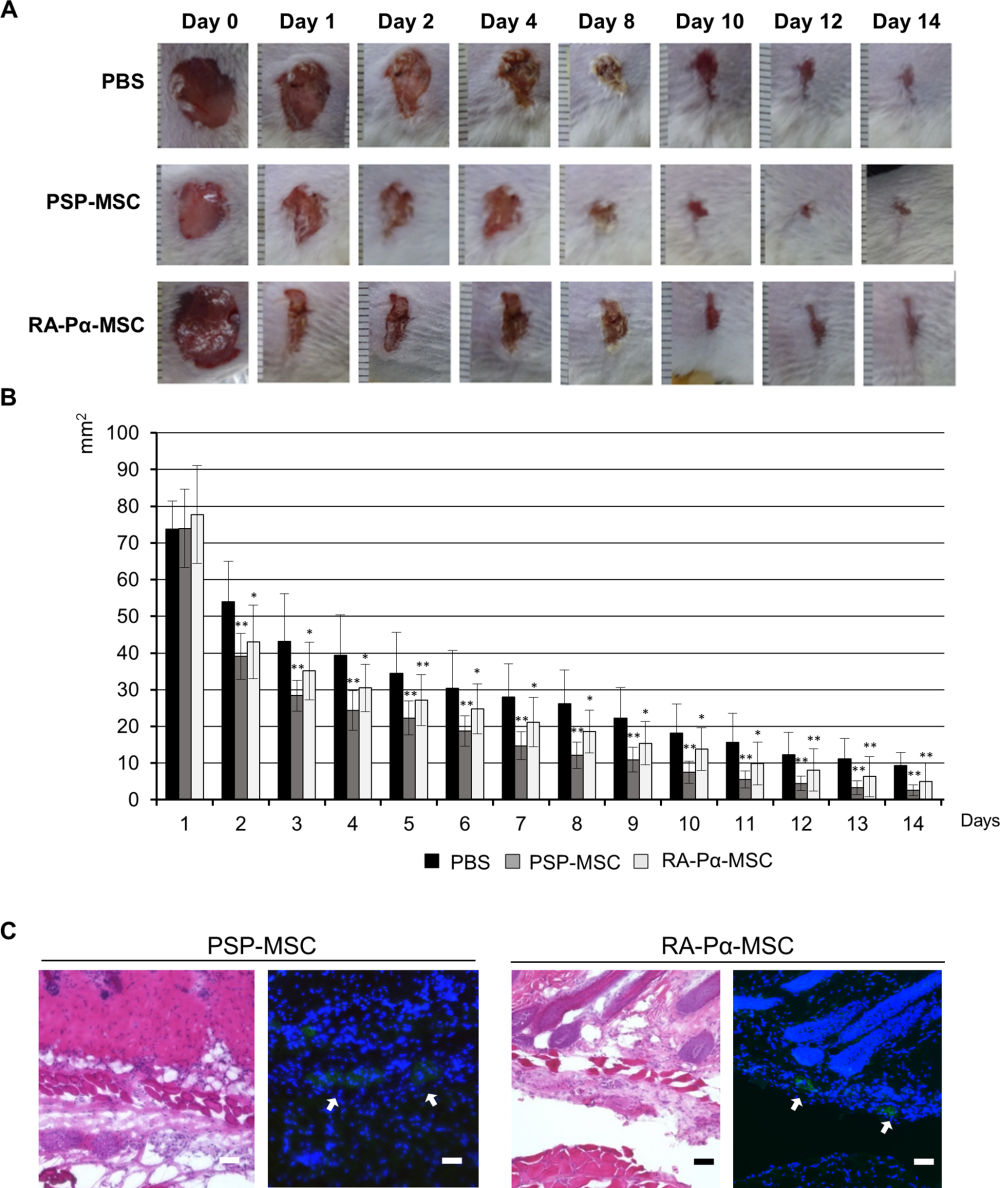
Treatments with PSP-MSC and RA-Pα-MSC on an in vivo skin-injury model of wound healing. (A, B): Representative photograph (A) and size of wound area (B) during the wound healing from day 0 to day 14 treated with PBS alone (n = 6), PSP-MSC (n = 6), and RA-Pα-MSC (n = 6). All experiments were conducted twice and representative data are shown. Data are means ± SDs. *P < 0.05, **P < 0.01, compared with PBS alone (t test). The divisions of scale are 1 mm (A). (C): Histological analysis of the wound edges in the day-14 mice treated with PSP-MSC and RA-Pα-MSC. Left panels are hematoxylin and eosin staining and right panels represent immunostaining with anti-human HLA antibodies. The arrows indicate the nest of PSP-MSC and RA-Pα-MSC stained with anti-human HLA antibodies (green) and DAPI for nuclei (blue). Scale bar = 40 μm.

We next asked whether PSP-MSCs and RA-Pα-MSCs show therapeutic potential using mouse models of human disease. Previous studies indicate that BM-MSCs could deliver new approaches to the management of wound healing in severe skin injuries [42]. To investigate whether PSP-MSCs and RA-Pα-MSCs could act similarly, we used a mouse model of wound healing generated by shaving the dorsal surfaces of immunodeficient mice and creating a full-thickness, 0.8-cm excision wound that extended to the fascia. The hiPSC-derived MSCs were then subcutaneously injected around each wound. We found that treatment with both hiPSC-derived MSCs accelerated the wound closure compared to control treatments, even in the early stages of wound healing. In this model, PSP-MSCs accelerated the wound healing faster than RA-Pα-MSCs (Fig 5A and 5B). Histological analysis revealed that the donor cells survived and aggregated to form a dermal nest around the wound edges, but not in the keratinocyte layer (Fig 5C).

**Fig 6:**
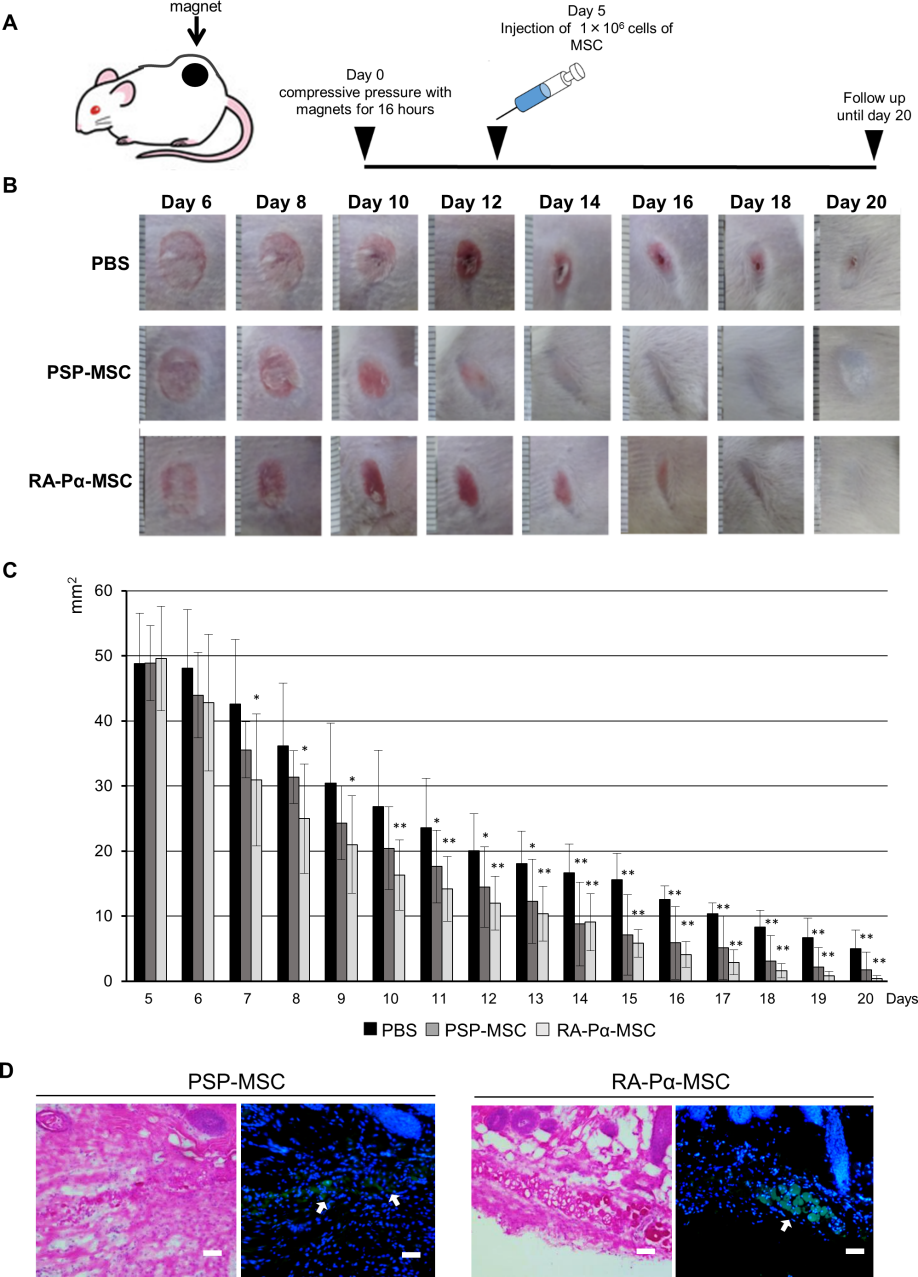
Treatments with PSP-MSC and RA-Pα-MSC on an in vivo pressure-induced skin ulcer model of wound healing. (A): Experimental design to generate the pressure-induced skin ulcer in mice. (B, C): Representative photograph (B) and size of wound area (C) during the wound healing from day 5 to day 20 treated with PBS alone (n = 8), PSP-MSC (n = 8), and RA-Pα-MSC (n = 8). All experiments were conducted twice and representative data are shown. Data are means ± SDs. *P < 0.05, **P < 0.01, compared with PBS alone (t test). The divisions of scale are 1 mm (B). (D): Histological analysis of the wound edges on day 20 from mice treated with PSP-MSC and RA-Pα-MSC. Left panels are hematoxylin and eosin staining and right panels represent immunostaining with anti-human HLA antibodies. The arrows indicate the nest of PSP-MSC and RA-Pα-MSC stained with anti-human HLA antibodies (green) and DAPI for nucleus (blue). Scale bar = 40 μm.

To further confirm the wound healing potential of our MSCs, we tested them in a second model of pressure ulcer in mouse skin (Fig 6A). The underlying mechanisms of ulcer formation differ between ulcer types, whereby a pressure ulcer develops due to ischemia in the skin tissue, while a skin ulcer (the first model tested herein) develops due to injury. To create skin ischemia, the dorsal skin of immunodeficient mice was pulled up and placed between two cylinders of magnets, producing a compressive pressure of 50 mmHg between the magnets (Fig 6A) [26]. After 16 hours of the ischemic pressure, the skin was reperfused and skin ulcers developed after 5 days. As described previously, we subcutaneously injected our MSCs around the ulcers. The MSC treatments also accelerated wound closure in this second ulcer model, compared to the control (Fig 6B and 6C), and similar to the first model, the donor cells survived and aggregated to form a nest in the dermis around the wound edges (Fig 6D). Notably, the RA-Pα-derived MSCs healed the ulcer faster than the PSP-derived MSCs, in an opposite effect to that observed with the first wound-healing model. These results indicate that our hiPSC-derived MSCs have therapeutic, but different effects on the two types of skin ulcer models in mice.

### hiPSC-derived MSCs can suppress cartilage defect in an osteoarthritis model

**Fig 7:**
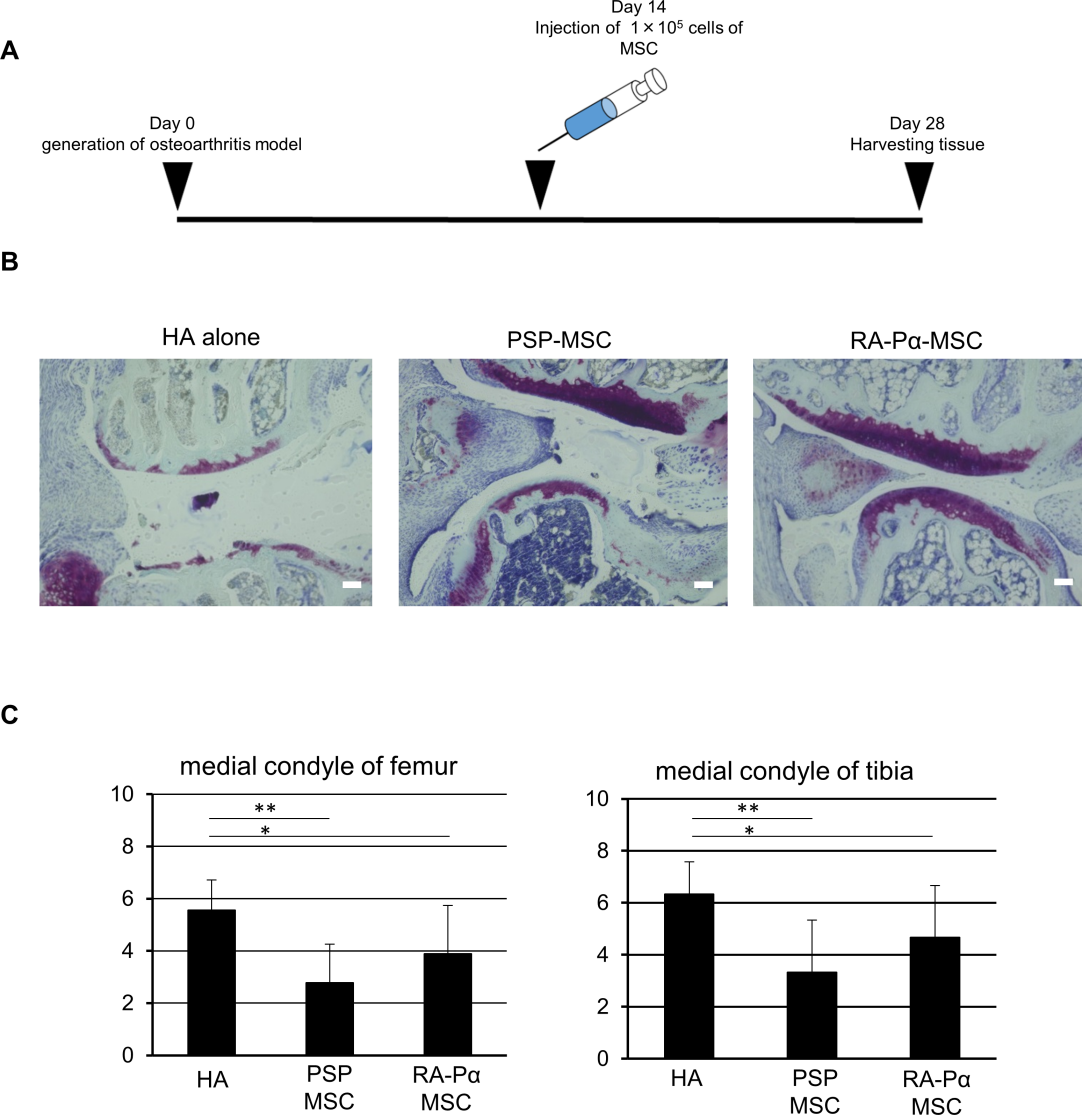
Suppressing degeneration of knee cartilage in the OA model mice treated with PSP-MSC and RA-Pα-MSC. (A): Experimental design to treat OA model mice with PSP-MSC and RA-Pα-MSC. On day 0, the anterior cruciate ligament (ACL) from each animal was transected, and then the medial meniscus was removed from the lower-limb joints. Two weeks later, PSP-MSC and RA-Pα-MSC were injected into the knee joint. On day 28, the treated knees were analyzed. (B): Histological analysis of knees in the OA model mice treated with HA alone, PSP-MSC, and RA-Pα-MSC on day 28 in sections stained with Safranin O. Scale bar = 20 μm. (C): Effect was evaluated by Modified Mankin scores in the OA model mice treated with HA (n = 9), PSP-MSC (n = 9), and RA-Pα-MSC (n = 9). Modified Mankin scores were measured by dyeability of the paracellular and intercellular regions, and the alignment of chondrocytes. The score is from 0 to 8 point. Data are means ± SDs. *P < 0.05, **P < 0.01, compared with HA alone (t test).

We next asked whether our MSCs could suppress cartilage degeneration and improve joint destruction in an osteoarthritis model (OA model), generated by transecting the anterior cruciate ligament (ACL), and then removing the medial meniscus from the lower limb joints of immunodeficient mice (Fig 7A) [27, 28]. We injected our MSCs dissolved with hyaluronic acids (HA) into the disrupted joints and asked whether they could suppress the defect. Histopathological analysis found that the joint cartilage treated with our MSCs plus HA was more effectively and significantly maintained than cartilage treated with HA alone (Fig 7B). Furthermore, the modified Mankin scores, which indicate the extent of joint cartilage damage [29], were also significantly improved in the mice treated with PSP- and RA-Pα-MSCs, compared with the mice treated with HA alone (Fig 7C); however, the effect of PSP-and RA-Pα-MSCs were similar in the OA model.

## Discussion

We directed human iPSCs to differentiate into MSCs based on the two distinct developmental processes, mesodermal and neuroepithelial differentiation. Several methods were recently developed for the direct differentiation of iPSCs into MSCs [12, 19, 43, 44]. In this study, we used a two-step method for the differentiation to MSCs from iPSCs. First, we generated PDGDRα+/VEGFR2- cells, which are MSC progenitor cells, purified by FACS after mesodermal and neuroepithelial differentiation. Next, we differentiated the sorted PDGDRα+/VEGFR2- cells into MSCs.

Our method has two advantages compared to those previously reported. First, the PDGDRα+/VEGFR2- cell purification step reduces contamination due to undifferentiated iPSCs. This is important in potential application of iPSCs for clinical medicine, because patients should be protected as much as possible from the risk of tumor formation due to contaminating undifferentiated cells, and a previous study demonstrated that PDGDRα- cells more frequently formed teratoma than PDGDRα+ cells [19]. In this study, transplanted PSP-MSCs and RA-Pα-MSCs did not form tumors in the testes of immunodeficient mice. The MSCs established herein are therefore expected to have a low potential for tumor formation. Second, our method can visualize and monitor the differentiation process with surface markers. This is useful for generating MSCs more stably and reliably. The differentiation potential is unstable in undifferentiated iPSCs and is easily affected by various factors such as passage number and culture condition. We sometimes experienced a low percentage of differentiated cells in vitro iPSC cultures even though the procedure of cell induction is identical to those previously used. The marker monitoring enables us to detect suspect points during the differentiation process in the in vitro iPSC culture. We purified two types of PDGDRα+/VEGFR2- cells from the in vitro iPSC cultures and induced them into MSCs. The PDGDRα+/VEGFR2- cells will be valuable for understanding not only the differentiation process from MSC progenitors to MSCs, but also the pathogenesis in cartilage and bone diseases such as fibrodysplasia ossificans progressiva and achondroplasia using the disease-derived iPSCs [45, 46].

We previously generated MSCs from mouse ES cells via neuroepithelial differentiation [2]. In this study, as like in the previous study, while *SOX1* expression was dramatically enhanced with RA treatment, *BRACHYURY* expression was dramatically reduced. In contrast, *SOX1* expression was markedly decreased during the mesodermal differentiation. In addition, the number of CFU-F in RA-Pα-MSCs was significantly higher than that in PSP-MSCs. Although some gene signatures in Fig 4E and 4F are similar between PSP-MSC and RA-Pα-MSC, the similarity of global gene expression is more dependent on the origin of MSCs. These results suggest that PSP-MSCs are different in gene identity from RA-Pα-MSCs.

To confirm the therapeutic effects of our MSCs, we tested both hiPSC-derived MSCs in three relevant mouse models of human diseases, namely skin wound healing, pressure sores, and osteoarthritis. Interestingly, the treatment with PSP-MSCs was more effective on the skin wound healing models than that with RA-Pα-MSCs. In contrast, the treatment with RA-Pα-MSCs more effectively cured the pressure ulcers than that with PSP-MSCs. Recent studies revealed that engrafted BM-MSCs expressed various paracrine factors with demonstrated therapeutic effects for several diseases [39–41]. The hierarchical clustering analysis of paracrine factors carried out in this study revealed the similarity between PSP-MSCs and the RA-Pα-MSCs; however, qPCR analysis revealed that HGF and EGF were more strongly expressed in PSP-derived MSCs than in the RA-Pα-derived MSCs. In contrast, RA-Pα-derived MSCs more highly expressed VEGF-A and bFGF than PSP-derived MSCs. HGF and EGF play a role in skin wound healing [47]. VEGF-A and bFGF play a role in angiogenesis, and the decreased expression of VEGF-A and bFGF leads to deterioration of pressure ulcer [48]. These factors thus elucidate the different effects observed herein between the two types of iPSC-derived MSCs in the tested disease models, and our results suggest that some therapeutic effects with MSCs are based on the potential for differential paracrine factor expression.

Toward the clinical application of iPSC-derived MSCs, we need to carefully select those iPSCs-derived MSCs that offer the best therapeutic potential for improving specific diseases. We previously demonstrated that neuroepithelium-derived MSCs disappear after birth in mice [2], and their isolation is difficult in adulthood. Our results now suggest that human neuroepithelium-derived MSCs have a different potential to treat diseases from mesoderm-derived MSCs, and that RA-Pα-derived MSCs are easily generated from iPSCs to compensate for this inconvenience. Allogenic transplantation is also preferred to autologous methods due to the ease and speed of patient treatment and the low cost of MSC stock preparation; however, a significant obstacle remaining with allogenic transplantation is immunological rejection due to unmatched HLA types between the donors and recipients [49]. We now propose overcoming this hurdle by preparing human iPSCs with the various HLA types and storing the resultant MSCs by a method such as that presented herein [50,51].

## Conclusion

In summary, we successfully generated MSCs from human iPSCs that showed both mesodermal differentiation and, by a novel procedure, neuroepithelial differentiation. We also showed the differential therapeutic potentials of these derived MSCs in various mice disease models. These findings suggest the exciting future prospect of selecting MSCs reservoirs and adapting procedures to generate MSCs from human iPSCs for treating specific disease conditions.

## Supporting information

S1 Fig Induction of hiPSC-derived MSCs under mesodermal and neuroepithelial differentiation conditions. (A): Proportion of PDGFRα and VEGFR2 expressions in day-6 differentiated 201B7 iPSC under the mesodermal differentiation condition. Numbers indicate the percentages of each population. (B): Effect of RA treatment on the proportions of PDGFRα and VEGFR2 in day-10 differentiated iPSCs under the neuroepithelial differentiation condition. The treatment periods are described for the upper panels. Numbers indicate the percentages of PDGFRα+/VEGFR2- (RA-Pα) population. Each experiment was conducted twice. (C): Proportions of PDGFRα and VEGFR2 expression in day-10 differentiated 201B7 iPSCs under the neuroepithelial differentiation condition. Numbers indicate the percentages of each population.
(TIF)

S2 Fig Immunofluprescence staining of MSC markers. Marker expressions of PSP-MSC (left) and RA-Pα-MSC (right). Scale bars: 20 μm.
(TIF)

S3 Fig Tumor formation activity. (A): Representative bright-field images of testes at 11 weeks after transplantations of iPSCs and iPSC-derived MSCs. N1-12 iPSC and 201B7 iPSC: testes transplanted with N1-12 (n = 2) and 201B7 iPSCs (n = 4), respectively. N1-12 PSP-MSC and RA-Pα-MSC: testes transplanted with N1-12-derived PSP-MSCs (n = 6) and RA-Pα-MSCs (n = 6), respectively. 201B7 PSP-MSC and RA-Pα-MSC: testes transplanted with 201B7-derived PSP-MSCs (n = 6) and RA-Pα-MSCs (n = 8), respectively. The size scale indicates centimeters (cm). (B and C): Histological analyses of testes in S3A Fig. Teratoma formation in the testes with the iPSC transplantations (B). Descendants from three germ layers were detected (B). CE: columnar epithelium (endoderm), C: cartilage (mesoderm), P: pigment cells (ectoderm). No tumor formation was detected in the testes transplanted with MSCs (C). All testes were examined by the histological analysis. Representative data of HE staining is shown. Scale bars: 40 μm.
(TIF)

S4 Fig DNA microarray analysis of PSP-MSC and RA-Pα-MSC. (A): Expression of pluripotent markers in N1-12 and 201B7 iPSCs by qPCR analysis. (B, C): Venn diagrams for data sets that were upregulated by 2.0-fold or more in PSP-MSC (B), or in RA-Pα-MSC (C), comparing to iPSC. The expressions of 286 data sets were commonly upregulated between N1-12-derived and 201B7-derived PSP-MSCs, and those of 359 data sets were commonly upregulated between N1-12-derived and 201B7-derived RA-Pα-MSCs. (D, E): Venn diagrams for data sets that were downregulated by 2.0-fold or more in PSP-MSC (D), or in RA-Pα-MSC (E), comparing to iPSC. The expressions of 221 data sets were commonly downregulated between N1-12-derived and 201B7-derived PSP-MSCs, and those of 178 data sets were commonly downregulated between N1-12-derived and 201B7-derived RA-Pα-MSCs. (F,G): Gene ontology (GO) analysis of 221 commonly downregulated data sets in PSP-MSC (F) and 178 data sets in RA-Pα-MSC (G). The top ten of GO terms are listed. GO terms were detected with a cutoff p-value of .1. Values are–log10 corrected p-value. Red color indicates different GO terms between (F) and (G).
(TIF)

S1 Table Primer list. (DOCX)

S2 Table Genes of pluripotent marker, MSC marker and paracrine factor. (DOCX)

## References

[1] Prockop DJ. Marrow stromal cells as stem cells for nonhematopoietic tissues. Science. 1997; 276: 71–74. 9082988 10.1126/science.276.5309.71

[2] Takashima Y, Era T, Nakano K, Kondo S, Kasuga M, Smith A.G, et al Neuroepithelial cells supply an initial transient wave of MSC differentiation. Cell. 2007; 129: 1377–1388. 10.1016/j.cell.2007.04.028 17604725

[3] Miwa H, Era T. Tracing the destiny of mesenchymal stem cells from embryo to adult bone marrow and white adipose tissue via Pdgfrα expression. Development. 2018; 29: 145. 10.1242/dev.155879 29378823

[4] Qi Y, Feng G, Yan W. Mesenchymal stem cell-based treatment for cartilage defects in osteoarthritis. Mol Biol Rep. 2012; 39: 5683–5689. 10.1007/s11033-011-1376-z 22183306

[5] Murphy WJ, Nolta JA. Autoimmune T cells lured to a FASL web of death of MSCs. Cell Stem Cell. 2012; 10: 485–487. 10.1016/j.stem.2012.04.013 22560070

[6] Resnick IB, Barkats C, Shapira M.Y, Stepensky P, Bloom A.I, Shimoni A, et al Treatment of severe steroid resistant acute GVHD with mesenchymal stromal cells (MSC). Am J Blood Res. 2013; 73: 225–238. PMC3755522 23997985

[7] Caplan AI, Correa D. The MSC: An Injury Drugstore. Cell Stem Cell. 2011; 9: 11–15. 10.1016/j.stem.2011.06.008 21726829 PMC3144500

[8] Tolar J, Le Blanc K, Keating A, Blazar B.R. Concise review: hitting the right spot with mesenchymal stromal cells. Stem Cells. 2010; 28: 1446–1455. 10.1002/stem.459 20597105 PMC3638893

[9] Hynes K, Menicanin D, Gronthos S, Bartold P M. Clinical utility of stem cells for periodontal regeneration. Periodontol 2000. 2012; 59: 203–227. 10.1111/j.1600-0757.2012.00443.x 22507067

[10] Wagner W, Horn P, Castoldi M, Diehlmann A, Bork S, Saffrich R, et al Replicative senescence of mesenchymal stem cells: A countinuous and organized process. Plos One. 2008; 21: e2213. 10.1371/journal.pone.0002213 PMC2374903 18493317

[11] Park IH, Zhao R, West JA, Yabuuchi A, Huo H, Ince T.A, et al Reprogramming of human somatic cells to pluripotency with defined factors. Nature. 2008; 451: 141–146. 10.1038/nature06534 18157115

[12] Zhang J, Guan J, Niu X, Hu G, Guo S, Li Q, et al Exosomes released from human induced pluripotent stem cells-derived MSCs facilitate cutaneous wound healing by promoting collagen synthesis and angiogenesis. J Transl Med. 2015; 13: 49 10.1186/s12967-015-0417-0 25638205 PMC4371881

[13] Bonab MM, Alimoghaddam K, Talebian F, Ghaffari S.H, Ghavamzadeh A, Nikbin B. Aging of mesenchymal stem cell in vitro. BMC Cell Biol. 2006; 7: 14 10.1186/1471-2121-7-14 16529651 PMC1435883

[14] Kang L, Wang J, Zhang Y, Kou Z, Gao S. iPS cells can support full-term development of tetraploid blastocyst-complemented embryos. Cell Stem Cell. 2009; 5: 135–138. 10.1016/j.stem.2009.07.001 19631602

[15] Hurley MM, Gronowicz G, Zhu L, Kuhn L.T, Rodner C, Xiao L. Age-related changes in FGF-2, fibroblast growth factor receptors and β-catenin expression in human mesenchyme-derived progenitor cells. J Cell Biochem. 2016; 117: 721–729. 10.1002/jcb.25357 26332075 PMC4861164

[16] Li Y, Charif N, Mainard D, Bensoussan D, Stoltz J F, de Isla N. Donor’s age dependent proliferation decrease of human bone marrow mesenchymal stem cells is linked to diminished clonogenicity. Biomed Mater Eng. 2014; 24: 47–52. 10.3233/BME-140973 24928917

[17] Nishimura T, Kaneko S, Kawana-Tachikawa A, Tajima Y, Goto H, Zhu D, et al Generation of rejuvenated antigen-specific T cells by reprogramming to pluripotency and redifferentiation. Cell Stem Cell. 2013; 12: 114–126. 10.1016/j.stem.2012.11.002 23290140

[18] Fukuta M, Nakai Y, Kirino K, Nakagawa M, Sekiguchi K, Nagata S, et al Derivation of mesenchyamal stromal cells from pluripotent stem cells through a neural crest lineage using small molecule compounds with defined media. Plos One. 2014; 9: e112291 10.1371/journal.pone.0112291 25464501 PMC4251837

[19] Sakurai H, Sakaguchi Y, Shoji E, Nishino T, Maki I, Sakai H, et al In vitro modeling of paraxial mesodermal progenitors derived from induced pluripotent stem cells. Plos One. 2012; 7: e47078 10.1371/journal.pone.0047078 23115636 PMC3480377

[20] Soga M, Ishitsuka Y, Hamasaki M, Yoneda K, Furuya H, Matuo M, et al HPGCD outperforms HPBCD as a potential treatment for Neimann-Pick disease type C during disease modeling with iPS cells. Stem Cells. 2014; 33: 1057–1088. 10.1002/stem.1917 25522247

[21] Sakurai H, Era T, Jakt LM, Okada M, Nakai S, Nishikawa S, et al In Vitro Modeling of Paraxial and Lateral Mesoderm Differentiation Reveals Early Reversibility. Stem Cells. 2006; 24: 575–586. 10.1634/stemcells.2005-0256 16339996

[22] Miwa H and Era T. Mesoderm Differentiation from hiPS Cells. Methods Mol Biol. 2014. 10.1007/7651_2014_162 25520286

[23] Okada S, Harada H, Ito T, Saito T, Suzu S. Early development of human hematopoietic and acquired immune systems in new born NOD/Scid/Jak3null mice intrahepatic engrafted with cord blood-derived CD34 + cells. Int J Hematol. 2008 12;88(5):476–482. 10.1007/s12185-008-0215-z 19039627

[24] Wysocki AB. Wound fluids and the pathogenesis of chronic wounds. J Wound Ostomy Continence Nurs. 1996; 23: 283–290. 9043277 10.1016/s1071-5754(96)90047-9

[25] Mishra PJ, Mishra PJ, Banerjee D. Cell-free derivatives from mesenchymal stem cells are effective in wound therapy. World J Stem Cells. 2012; 4: 35–43. 10.4252/wjsc.v4.i5.35 22993660 PMC3443710

[26] Kasuya A, Sakabe J, Tokura Y. Potential application of in vivo imaging of impaired lymphatic duct to evaluate the severity of pressure ulcer in mouse model. Sci Rep. 2014; 4: 4173 10.1038/srep04173 24566895 PMC3933905

[27] Christiansen BA, Guilak F, Lockwood KA, Olson S.A, Pitsillides A.A, Sandell L.J, et al Non-invasive mouse models of post-traumatic osteoarthritis. Osteoarthritis Cartilage. 2015; 23: 1627–1638. 10.1016/j.joca.2015.05.009 26003950 PMC4577460

[28] Horie M, Sekiya I, Muneta T, Ichinose S, Matsumoto K, Saito H, et al Intra-articular injected synovial stem cells differentiate into meniscal cells directly and promote meniscal regeneration without mobilization to distant organs in rat massive meniscal defect. Stem Cells. 2009; 27: 878–887. 10.1634/stemcells.2008-0616 19350690

[29] Bomsta BD, Bridgewater LC, Seegmiller RE. Premature osteoarthritis in the Disproportionate micromelia (Dmm) mouse. Osteoarthritis Cartilage. 2006; 14: 477–485. 10.1016/j.joca.2005.11.011 16431140

[30] D'Antonio M, Woodruff G, Nathanson JL, D’Antonio-Chronowska A, Arias A, Matsui H, et al High-Throughput and Cost-Effective Characterization of Induced Pluripotent Stem Cells. Stem Cell Reports. 2017; 11: 1101–1111. 10.1016/j.stemcr.2017.03.011 PMC5390243 28410643

[31] Pripuzova NS, Getie-Kebtie M, Grunseich C, Sweeney C, Malech H, Alterman M.A. Development of a protein marker panel for characterization of human induced pluripotent stem cells (hiPSCs) using global quantitative proteome analysis. Stem Cell Res. 2015; 14: 323–38. 10.1016/j.scr.2015.01.009 25840413 PMC5778352

[32] Takahashi K, Tanabe K, Ohnuki M, Narita M, Ichisaka T, Tomoda K, et al Induction of pluripotent stem cells from adult human fibroblasts by defined factors. Cell. 2007; 131: 861–872. 10.1016/j.cell.2007.11.019 18035408

[33] Bühring HJ, Treml S, Cerabona F, de Zwart P, Kanz L, Sobiesiak M. Phenotypic characterization of distinct human bone marrow-derived MSC subsets. Ann N Y Acad Sci. 2009; 1176: 124–134. 10.1111/j.1749-6632.2009.04564.x 19796240

[34] Pittenger MF, Martin BJ. Mesenchymal stem cells and their potential as cardiac therapeutics. Circ Res. 2004; 9: 9–20. 10.1161/01.RES.0000135902.99383.6f 15242981

[35] Samsonraj RM, Raghunath M, Nurcombe V, Hui JH, van Wijnen AJ, Cool SM. Concise Review: Multifaceted Characterization of Human Mesenchymal Stem Cells for Use in Regenerative Medicine. Stem Cells Transl Med. 2017; 6: 2173–2185. 10.1002/sctm.17-0129 29076267 PMC5702523

[36] Al-Nbaheen M, Vishnubalaji R, Ali D, Bouslimi A, AI-Jassir F, Megges M, et al Human stromal (mesenchymal) stem cells from bone marrow, adipose tissue and skin exhibit differences in molecular phenotype and differentiation potential. Stem Cell Rev. 2013; 9: 32–43. 10.1007/s12015-012-9365-8 22529014 PMC3563956

[37] Kyurkchiev D, Bochev I, Ivanova-Todorova E, Mourdjeva M, Oreshkova T, Belemezova K, et al Secretion of immunoregulatory cytokines by mesenchymal stem cells. World J Stem Cells. 2014; 26: 552–570. 10.4252/wjsc.v6.i5.552 PMC4178255 25426252

[38] Liang X, Ding Y, Zhang Y, Tse H.F, Lian Q. Paracrine mechanisms of mesenchymal stem cell-based therapy: current status and perspectives. Cell Transplant. 2014; 23: 1045–1059. 10.3727/096368913X667709 23676629

[39] Mirotsou M, Jayawardena TM, Schmeckpeper J, Gnecchi M, Dazu V.J. Paracrine mechanisms for stem cell reparative and regenerative actions in the heart. J Mol Cell Cardiol. 2011; 50: 280–289. 10.1016/j.yjmcc.2010.08.005 20727900 PMC3021634

[40] Bortolotti F, Ukovich L, Razban V, Martinelli V, Ruozi G, Pelos S, et al In vivo therapeutic potential of mesenchymal stromal cells depends on the source and the isolation procedure. Stem Cell Reports. 2015; 4: 322–329. 10.1016/j.stemcr.2015.01.001 PMC4375942 25660405

[41] Gnecchi M, Danieli P, Cervio E. Mesenchymal stem cell therapy for heart disease. Vascul Pharmacol. 2012; 57: 48–55. 10.1016/j.vph.2012.04.002 22521741

[42] Basiouny HS, Salama NM, Maadawi ZM, Farag E.A. Effect of bone marrow derived mesenchymal stem cells on healing of induced full-thickness skin wounds in albino rat. Int J Stem Cells. 2013; 6: 12–25. 24298370 10.15283/ijsc.2013.6.1.12 PMC3840998

[43] Sheyn D, Ben-David S, Shapiro G, de Mel S, Bez M, Ornelas L, et al Human induced pluripotent stem cells differentiate into functional mesenchymal stem cells and repair bone defects. Stem Cells Transl Med. 2016; 5: 1447–1460. 10.5966/sctm.2015-0311 27400789 PMC5070500

[44] Villa-Diaz LG, Brown SE, Liu Y, Ross A.M, Lahann J, Parent J.M, et al Derivation of mesenchymal stem cells from human induced pluripotent stem cells cultured on synthetic substrates. Stem Cells. 2012; 30: 1174–1181. 10.1002/stem.1084 22415987 PMC3549569

[45] Hamasaki M, Hashizume Y, Yamada Y, Katayama T, Honjoh H, Fusaki N, et al Pathogenic Mutation of ALK2 Inhibits Induced Pluripotent Stem Cell Reprogramming and Maintenance: Mechanisms of Reprogramming and Strategy for Drug Identification. Stem Cells. 2012; 30: 2437–2449. 10.1002/stem.1221 22949078

[46] Yamashita A, Morioka M, Kishi H, Kimura T, Yahara Y, Okada M, et al Statin treatment rescues FGFR3 skeletal dysplasia phenotypes. Nature. 2014; 513: 507–511. 10.1038/nature13775 25231866

[47] Li JF, Duan HF, Wu CT, Zhang DJ, Deng Y, Yin HL, et al HGF accelerates wound healing by promoting the dedifferentiation of epidermal cells through β1-integrin/ILK pathway. Biomed Res Int. 2013; 2013:470418 10.1155/2013/470418 24490163 PMC3899705

[48] Yang JJ, Wang XL, Shi BW, Huang F. The angiogenic peptide vascular endothelial growth factor-basic fibroblast growth factor signaling is up-regulated in a rat pressure ulcer model. Anat Rec (Hoboken). 2013; 296:1161–8. 23740668 10.1002/ar.22676

[49] Ankrum JA, Ong JF, Karp JM. Mesenchymal stem cells: immune evasive, not immune privileged. Nat Biotechnol. 2014; 32: 252–260. 10.1038/nbt.2816 24561556 PMC4320647

[50] de Rham C, Villard J. Potential and limitation of HLA-based banking of human pluripotent stem cells for cell therapy. J Immunol Res. 2014; 2014: 518135 10.1155/2014/518135 25126584 PMC4121106

[51] Van Essen TH, Roelen DL, Williams KA, Jager MJ. Matching for Human Leukocyte Antigens (HLA) in corneal transplantation—to do or not to do. Prog Retin Eye Res. 2015; 46: 84–110. 10.1016/j.preteyeres.2015.01.001 25601193

