# Supplementary figures and images for: Mesenchymal stem cells derived from human iPS cells via mesoderm and neuroepithelium have different features and therapeutic potentials
Source: PLoS One. 2018 Jul 25;13(7):e0200790. doi: 10.1371/journal.pone.0200790 (PMC6059447; doi:10.1371/journal.pone.0200790)

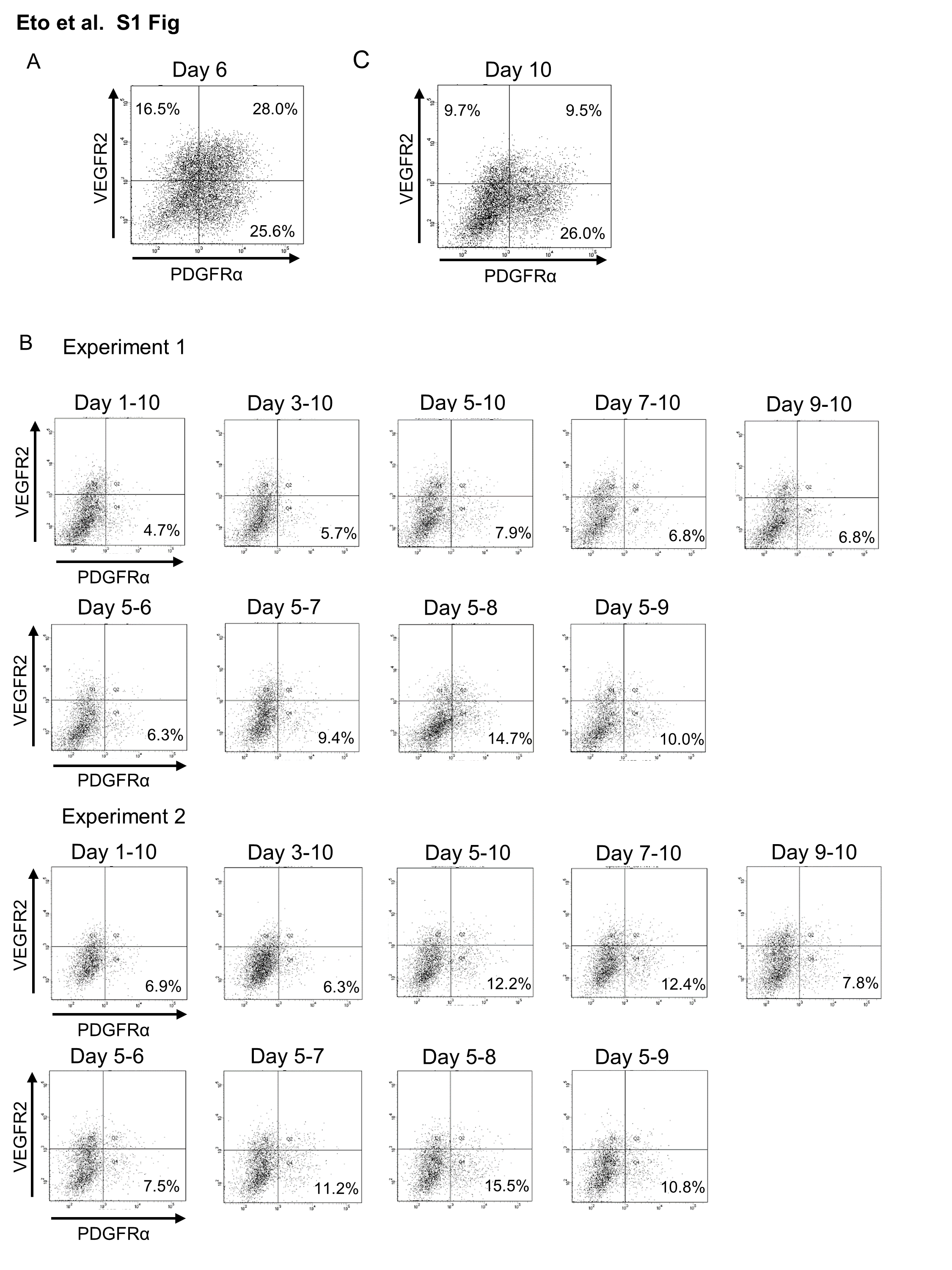

Supplement: S1 Fig — (A): Proportion of PDGFRα and VEGFR2 expressions in day-6 differentiated 201B7 iPSC under the mesodermal differentiation condition. Numbers indicate the percentages of each population. (B): Effect of RA treatment on the proportions of PDGFRα and VEGFR2 in day-10 differentiated iPSCs under the neuroepithelial differentiation condition. The treatment periods are described for the upper panels. Numbers indicate the percentages of PDGFRα+/VEGFR2- (RA-Pα) population. Each experiment was conducted twice. (C): Proportions of PDGFRα and VEGFR2 expression in day-10 differentiated 201B7 iPSCs under the neuroepithelial differentiation condition. Numbers indicate the percentages of each population. (TIF) [file pone.0200790.s001.TIF]

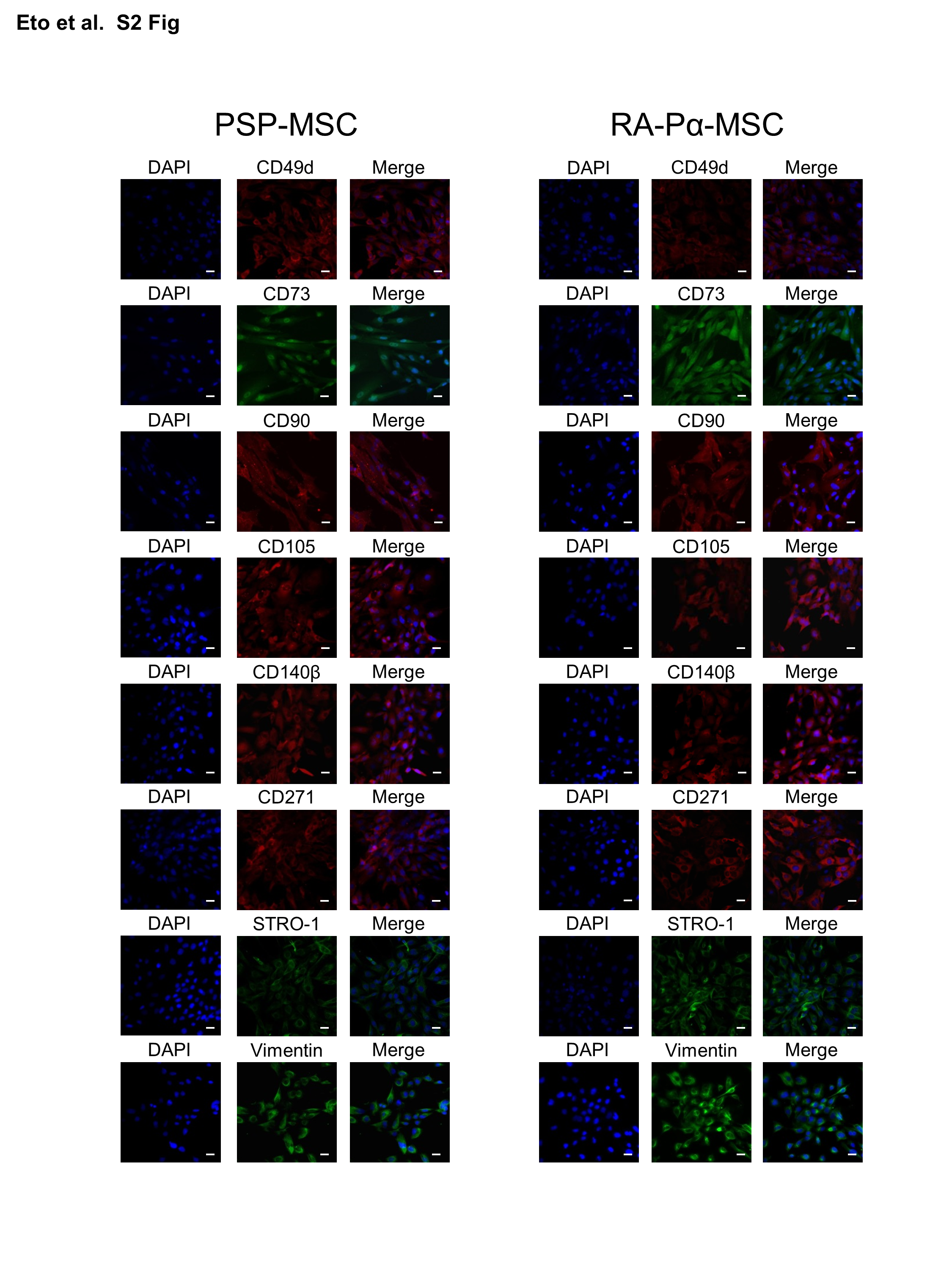

Supplement: S2 Fig — Marker expressions of PSP-MSC (left) and RA-Pα-MSC (right). Scale bars: 20 μm. (TIF) [file pone.0200790.s002.TIF]

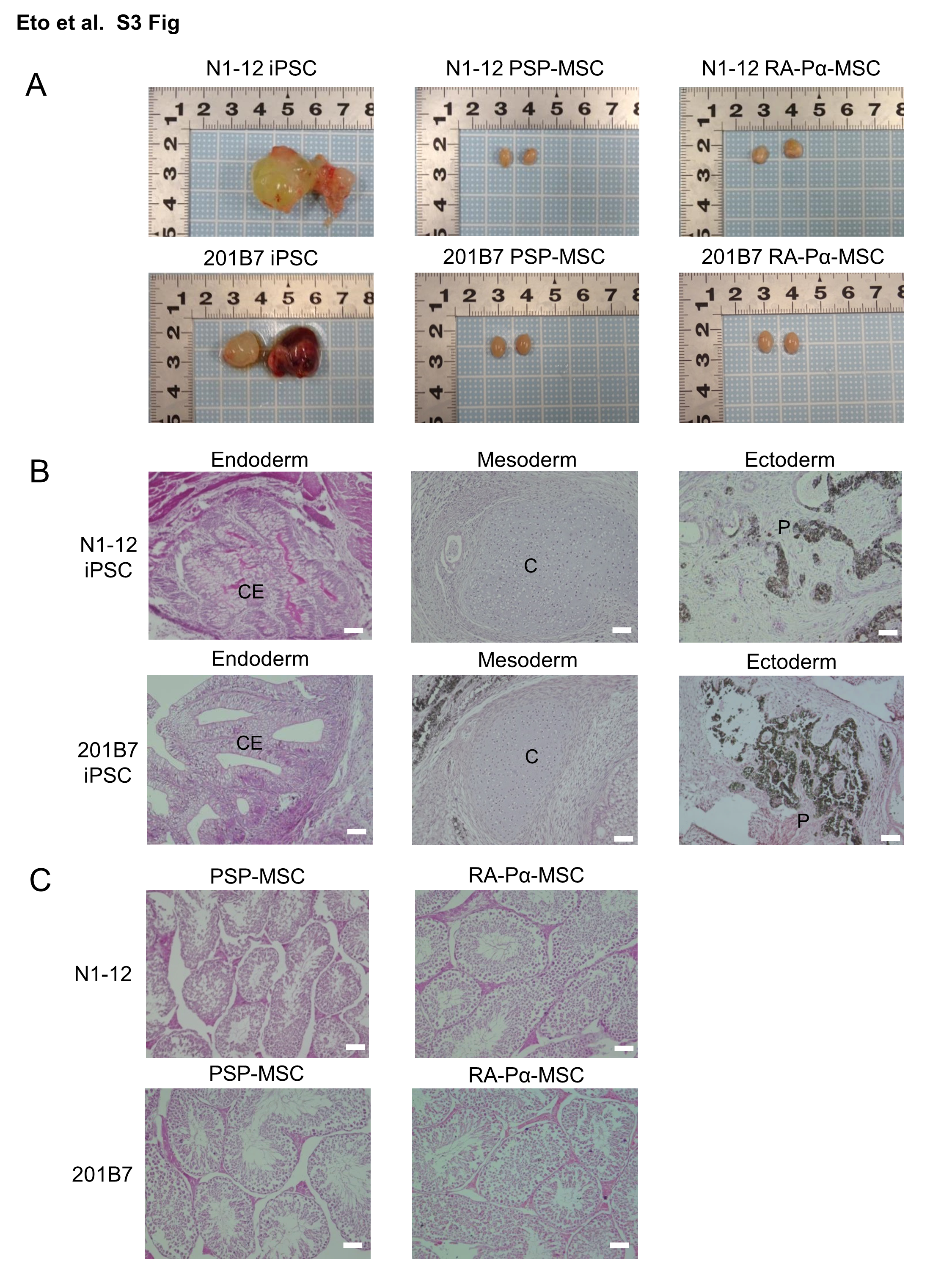

Supplement: S3 Fig — (A): Representative bright-field images of testes at 11 weeks after transplantations of iPSCs and iPSC-derived MSCs. N1-12 iPSC and 201B7 iPSC: testes transplanted with N1-12 (n = 2) and 201B7 iPSCs (n = 4), respectively. N1-12 PSP-MSC and RA-Pα-MSC: testes transplanted with N1-12-derived PSP-MSCs (n = 6) and RA-Pα-MSCs (n = 6), respectively. 201B7 PSP-MSC and RA-Pα-MSC: testes transplanted with 201B7-derived PSP-MSCs (n = 6) and RA-Pα-MSCs (n = 8), respectively. The size scale indicates centimeters (cm). (B and C): Histological analyses of testes in S3A Fig. Teratoma formation in the testes with the iPSC transplantations (B). Descendants from three germ layers were detected (B). CE: columnar epithelium (endoderm), C: cartilage (mesoderm), P: pigment cells (ectoderm). No tumor formation was detected in the testes transplanted with MSCs (C). All testes were examined by the histological analysis. Representative data of HE staining is shown. Scale bars: 40 μm. (TIF) [file pone.0200790.s003.TIF]

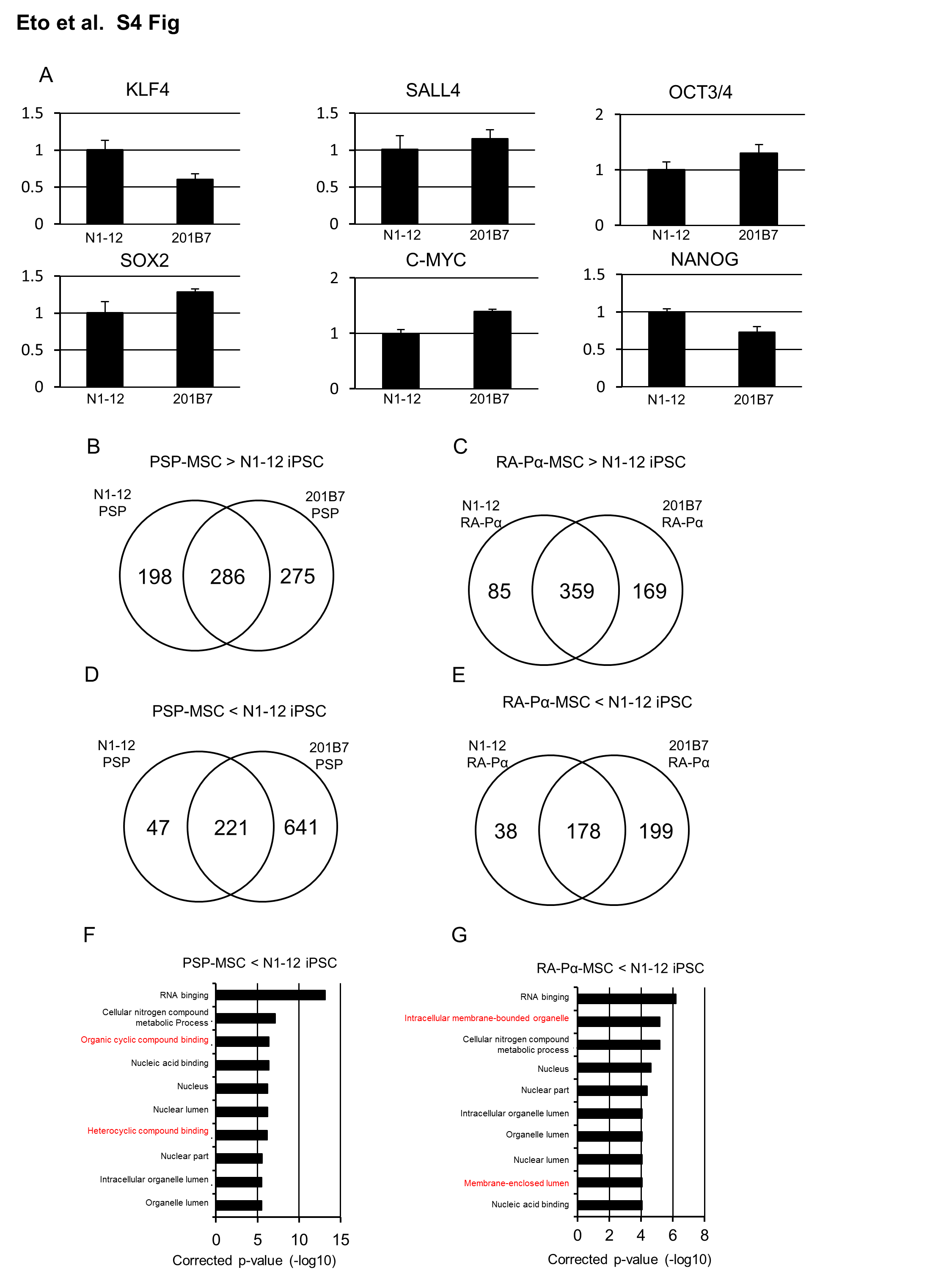

Supplement: S4 Fig — (A): Expression of pluripotent markers in N1-12 and 201B7 iPSCs by qPCR analysis. (B, C): Venn diagrams for data sets that were upregulated by 2.0-fold or more in PSP-MSC (B), or in RA-Pα-MSC (C), comparing to iPSC. The expressions of 286 data sets were commonly upregulated between N1-12-derived and 201B7-derived PSP-MSCs, and those of 359 data sets were commonly upregulated between N1-12-derived and 201B7-derived RA-Pα-MSCs. (D, E): Venn diagrams for data sets that were downregulated by 2.0-fold or more in PSP-MSC (D), or in RA-Pα-MSC (E), comparing to iPSC. The expressions of 221 data sets were commonly downregulated between N1-12-derived and 201B7-derived PSP-MSCs, and those of 178 data sets were commonly downregulated between N1-12-derived and 201B7-derived RA-Pα-MSCs. (F,G): Gene ontology (GO) analysis of 221 commonly downregulated data sets in PSP-MSC (F) and 178 data sets in RA-Pα-MSC (G). The top ten of GO terms are listed. GO terms were detected with a cutoff p-value of .1. Values are–log10 corrected p-value. Red color indicates different GO terms between (F) and (G). (TIF) [file pone.0200790.s004.TIF]
