# Supplementary material for: Mesenchymal stem cells derived from human iPS cells via mesoderm and neuroepithelium have different features and therapeutic potentials
Source: PLoS One. 2018 Jul 25;13(7):e0200790. doi: 10.1371/journal.pone.0200790 (PMC6059447; doi:10.1371/journal.pone.0200790)
Supplement: S1 Table — (DOCX) [file pone.0200790.s005.docx]

**S1 Table. Primer list**

| Primer | sequence |
| --- | --- |
| GAPDH-forward | 5'-GGCTGGCATTGATTGCCCTCAACG-3' |
| GAPDH-reverse | 5'-AGGGACTCCCCAGCAGTGAG-3' |
| NANOG-forward | 5’-CCAAAGGCAAACAACCCACTT-3’ |
| NANOG-reverse | 5’-CGGGACCTTGTCTTCCTTTTT-3’ |
| OCT3/4-forward | 5’-GGAAGGAATTGGGAACACAAAGG-3’ |
| OCT3/4-reverse | 5’-AACTTCACCTTCCCTCCAACCA-3’ |
| VIMENTIN-forward | 5’-CGTGAATACCAAGACCTGCTC-3’ |
| VIMENTIN-reverse | 5’-GGAAAAGTTTGGAAGAGGCAG-3’ |
| PDGFRβ-forward | 5’-CAATGTGACGGAGAGTGTGAATG-3’ |
| PDGFRβ-reverse | 5’-ACGTAGCCGCTCTCAACCA-3’ |
| SOX1-forward | 5’-GCCCTGAGCCGACTGTGA-3’ |
| SOX1-reverse | 5’-CCGTGAATACGATGAGTG-3’ |
| BRACHYURY-forward | 5’-GGTGTGAGCGTGCAGCTAGA-3’ |
| BRACHYURY-reverse | 5’-TGCCCAGCTGGTTGAACTC-3’ |
| BMP2-forward | 5'-CTATCAGGACATGGTTGTGGAG-3' |
| BMP2-reverse | 5'-GGGAAATATTAAAGTGTCAACTGGG-3' |
| TGFB1-forward | 5'-GCCTTTCCTGCTTCTCATGG-3' |
| TGFB1-reverse | 5'-TCCTTGCGGAAGTCAATGTAC-3' |
| HGF-forward | 5'-GCTATACTCTTGACCCTCACAC-3' |
| HGF-reverse | 5'-GTAGCCTTCTCCTTGACCTTG-3' |
| VFGFA-forward | 5'-AGGGCAGAATCATCACGAAG-3' |
| VEGFA-reverse | 5'-GGATGGCTTGAAGATGTACTCG-3' |
| EGF-forward | 5'-GATGGTTCAAAACGCCGAAG-3' |
| EGF-reverse | 5'-CGTACTCTATCTTTGCCAGTCC-3' |
| bFGF-forward | 5'-ACCCTCACATCAAGCTACAAC-3' |
| bFGF-reverse | 5'-AAAAGAAACACTCATCCGTAACAC-3' |
| PDGFβ-forward | 5'-AGTCGGCATGAATCGCTG-3' |
| PDGFβ-reverse | 5'-CATCAAAGGAGCGGATCGAG-3' |
| SOX9-forward | 5'-AGATGTGCGTCTGCTCCGTG-3' |
| SOX9-reverse | 5'-AGGTGCTCAAAGGCTACGACT-3' |
| AGGRECAN-forward | 5'-ACTTGCGTCTACCCCAATCC-3' |
| AGGRECAN-reverse | 5'-ACAGTCTTGCCCCACTTACC-3' |
| RUNX2-forward | 5'-AGCAAGGTTCAACGATCTGAG-3' |
| RUNX2-reverse | 5'-TGAAGACGGTTATGGTCAAGG-3' |
| OCN-forward | 5'-CACACTCCTCGCCCTATTG-3' |
| OCN-reverse | 5'-GTCTCTTCACTACCTCGCTG-3' |
| PPARr-forward | 5'-AGCCTCATGAAGAGCCTTCCA-3' |
| PPARr-reverse | 5'-ACCCTTGCATCCTTCACAAGC-3' |
| FABP4-forward | 5'-GCATGGCCAAACCTAACATGA-3' |
| FABP4-reverse | 5'-CCTGGCCCAGTATGAAGGAAA-3' |
| KLF4-forward | 5’-ACCTACACAAAGAGTTCCCATC-3’ |
| KLF4-reverse | 5’-TGTGTTTACGGTAGTGCCTG-3’ |
| SALL4-forward | 5’-GCCGAAAGCATCAAGTCAAAG-3’ |
| SALL4-reverse | 5’-GATAAACGTGGAAGGGAGACTG-3’ |
| SOX2-forward | 5’-ACAGCAAATGACAGCTGCAAA-3’ |
| SOX2-reverse | 5’-TCGGCATCGCGGTTTTT-3’ |
| C-MYC-forward | 5’-GCTTCTCTGAAAGGCTCTCC-3’ |
| C-MYC-reverse | 5’-AGTAGAAATACGGCTGCACC-3’ |
