# Supplementary material for: Mesenchymal stem cells derived from human iPS cells via mesoderm and neuroepithelium have different features and therapeutic potentials
Source: PLoS One. 2018 Jul 25;13(7):e0200790. doi: 10.1371/journal.pone.0200790 (PMC6059447; doi:10.1371/journal.pone.0200790)
Supplement: S2 Table — (DOCX) [file pone.0200790.s006.docx]

**S2 Table. Genes of pluripotent marker, MSC marker and paracrine factor**

| gene | marker | reference |
| --- | --- | --- |
| oct3/4 | pluripotent | Takahashi et al., 2007 |
| sox2 | pluripotent | Takahashi et al., 2007 |
| nanog | pluripotent | Takahashi et al., 2007 |
| GDF3 | pluripotent | Takahashi et al., 2007 |
| REX1 | pluripotent | Takahashi et al., 2007 |
| FGF4 | pluripotent | Takahashi et al., 2007 |
| ESG1 | pluripotent | Takahashi et al., 2007 |
| DPPA2 | pluripotent | Takahashi et al., 2007 |
| DPPA4 | pluripotent | Takahashi et al., 2007 |
| hTERT | pluripotent | Takahashi et al., 2007 |
| DNMT3B | pluripotent | Takahashi et al., 2007 |
| GABRB3 | pluripotent | Takahashi et al., 2007 |
| TDGF1 | pluripotent | Takahashi et al., 2007 |
| GAL | pluripotent | Takahashi et al., 2007 |
| LEFTB | pluripotent | Takahashi et al., 2007 |
| IFITM1 | pluripotent | Takahashi et al., 2007 |
| NODAL | pluripotent | Takahashi et al., 2007 |
| UTF1 | pluripotent | Takahashi et al., 2007 |
| EBAF | pluripotent | Takahashi et al., 2007 |
| GRB7 | pluripotent | Takahashi et al., 2007 |
| PODXL | pluripotent | Takahashi et al., 2007 |
| CD9 | pluripotent | Takahashi et al., 2007 |
| BRIX | pluripotent | Takahashi et al., 2007 |
| KLF4 | pluripotent | Takahashi et al., 2007 |
| c-MYC | pluripotent | Takahashi et al., 2007 |
| NAT1 | pluripotent | Takahashi et al., 2007 |
| SALL4 | pluripotent | Takahashi et al., 2007 |
| FOXD3 | pluripotent | Takahashi et al., 2007 |
| E-CADHERIN | pluripotent | Takahashi et al., 2007 |
| TRA1-60 | pluripotent | Takahashi et al., 2007 |
| AP | pluripotent | Takahashi et al., 2007 |
| TRA1-81 | pluripotent | Takahashi et al., 2007 |
| SSEA1 | pluripotent | Takahashi et al., 2007 |
| SSEA3 | pluripotent | Takahashi et al., 2007 |
| SSEA4 | pluripotent | Takahashi et al., 2007 |
| TRA-2-49 | pluripotent | Takahashi et al., 2007 |
| CHD1 | pluripotent | D'Antonio et al., 2017 |
| CHD7 | pluripotent | D'Antonio et al., 2017 |
| FUT4 | pluripotent | D'Antonio et al., 2017 |
| UTF1 | pluripotent | D'Antonio et al., 2017 |
| KAT5 | pluripotent | D'Antonio et al., 2017 |
| THAP11 | pluripotent | D'Antonio et al., 2017 |
| ZFP42 | pluripotent | D'Antonio et al., 2017 |
| DPPA3 | pluripotent | D'Antonio et al., 2017 |
| DPPA5 | pluripotent | D'Antonio et al., 2017 |
| MYBL2 | pluripotent | D'Antonio et al., 2017 |
| NR5A2 | pluripotent | D'Antonio et al., 2017 |
| RIF1 | pluripotent | D'Antonio et al., 2017 |
| SCN1A | pluripotent | D'Antonio et al., 2017 |
| SOX15 | pluripotent | D'Antonio et al., 2017 |
| CDC42 | pluripotent | D'Antonio et al., 2017 |
| CDK1 | pluripotent | D'Antonio et al., 2017 |
| AIBP | pluripotent | Pripuzova et al., 2015 |
| APOE | pluripotent | Pripuzova et al., 2015 |
| CTNNB1 | pluripotent | Pripuzova et al., 2015 |
| MFGF | pluripotent | Pripuzova et al., 2015 |
| NEST | pluripotent | Pripuzova et al., 2015 |
| CXADR | pluripotent | Pripuzova et al., 2015 |
| FUBP3 | pluripotent | Pripuzova et al., 2015 |
| GPC4 | pluripotent | Pripuzova et al., 2015 |
| HDAC2 | pluripotent | Pripuzova et al., 2015 |
| HDGF | pluripotent | Pripuzova et al., 2015 |
| IGF2BP1 | pluripotent | Pripuzova et al., 2015 |
| LIN28 | pluripotent | Pripuzova et al., 2015 |
| TER1 | pluripotent | Pripuzova et al., 2015 |
| CD10 | MSC | Bühring et al., 2009 |
| CD13 | MSC | Samsonraj et al., 2017 |
| CD15 | MSC | Al-Nbaheen et al., 2013 |
| CD18 | MSC | Al-Nbaheen et al., 2013 |
| CD29 | MSC | Samsonraj et al., 2017 |
| CD44 | MSC | Samsonraj et al., 2017 |
| CD49a | MSC | Samsonraj et al., 2017 |
| CD49b | MSC | Samsonraj et al., 2017 |
| CD49c | MSC | Samsonraj et al., 2017 |
| CD49e | MSC | Samsonraj et al., 2017 |
| CD49f | MSC | Pittenger et al., 2003 |
| CD50 | MSC | Al-Nbaheen et al., 2013 |
| CD51 | MSC | Samsonraj et al., 2017 |
| CD54 | MSC | Samsonraj et al., 2017 |
| CD58 | MSC | Samsonraj et al., 2017 |
| CD61 | MSC | Samsonraj et al., 2017 |
| CD63 | MSC | Al-Nbaheen et al., 2013 |
| CD71 | MSC | Al-Nbaheen et al., 2013 |
| CD73 | MSC | Al-Nbaheen et al., 2013 |
| CD82 | MSC | Al-Nbaheen et al., 2013 |
| CD90 | MSC | Al-Nbaheen et al., 2013 |
| CD97 | MSC | Al-Nbaheen et al., 2013 |
| CD98 | MSC | Al-Nbaheen et al., 2013 |
| CD99 | MSC | Al-Nbaheen et al., 2013 |
| CD102 | MSC | Pittenger et al., 2003 |
| CD103 | MSC | Al-Nbaheen et al., 2013 |
| CD105 | MSC | Al-Nbaheen et al., 2013 |
| CD106 | MSC | Al-Nbaheen et al., 2013 |
| CD112 | MSC | Al-Nbaheen et al., 2013 |
| CD113 | MSC | Al-Nbaheen et al., 2013 |
| CD119 | MSC | Al-Nbaheen et al., 2013 |
| CD120b | MSC | Samsonraj et al., 2017 |
| CD120a | MSC | Samsonraj et al., 2017 |
| CD124 | MSC | Samsonraj et al., 2017 |
| CD140a | MSC | Samsonraj et al., 2017 |
| CD140b | MSC | Samsonraj et al., 2017 |
| CD146 | MSC | Bühring et al., 2009 |
| CD166 | MSC | Bühring et al., 2009 |
| CD200 | MSC | Bühring et al., 2009 |
| CD271 | MSC | Samsonraj et al., 2017 |
| HLA-A | MSC | Pittenger et al., 2003 |
| HLA-B | MSC | Pittenger et al., 2003 |
| HLA-C | MSC | Pittenger et al., 2003 |
| COL1A1 | MSC | Pittenger et al., 2003 |
| COL3A1 | MSC | Pittenger et al., 2003 |
| IFNr | paracrine factor | Kyurkchiev et al., 2014 |
| TNFa | paracrine factor | Kyurkchiev et al., 2014 |
| IL-1b | paracrine factor | Kyurkchiev et al., 2014 |
| PGE2 | paracrine factor | Kyurkchiev et al., 2014 |
| ICAM | paracrine factor | Kyurkchiev et al., 2014 |
| VEGF | paracrine factor | Kyurkchiev et al., 2014 |
| IDO | paracrine factor | Kyurkchiev et al., 2014 |
| TGFb | paracrine factor | Kyurkchiev et al., 2014 |
| IL-10 | paracrine factor | Kyurkchiev et al., 2014 |
| IL-6 | paracrine factor | Kyurkchiev et al., 2014 |
| IDO | paracrine factor | Kyurkchiev et al., 2014 |
| ICAM | paracrine factor | Kyurkchiev et al., 2014 |
| PGE2 | paracrine factor | Kyurkchiev et al., 2014 |
| CCL2 | paracrine factor | Kyurkchiev et al., 2014 |
| CCL3 | paracrine factor | Kyurkchiev et al., 2014 |
| CCL4 | paracrine factor | Kyurkchiev et al., 2014 |
| CCL5 | paracrine factor | Kyurkchiev et al., 2014 |
| CCL7 | paracrine factor | Kyurkchiev et al., 2014 |
| CCL20 | paracrine factor | Kyurkchiev et al., 2014 |
| CCL26 | paracrine factor | Kyurkchiev et al., 2014 |
| CXCL1 | paracrine factor | Kyurkchiev et al., 2014 |
| CXCL2 | paracrine factor | Kyurkchiev et al., 2014 |
| CXCL5 | paracrine factor | Kyurkchiev et al., 2014 |
| CXCL8 | paracrine factor | Kyurkchiev et al., 2014 |
| CXCL10 | paracrine factor | Kyurkchiev et al., 2014 |
| CXCL11 | paracrine factor | Kyurkchiev et al., 2014 |
| CXCL12 | paracrine factor | Kyurkchiev et al., 2014 |
| CX3CL1 | paracrine factor | Kyurkchiev et al., 2014 |
| SFRP2 | paracrine factor | Mirotsou et al., 2011 |
| HGF | paracrine factor | Mirotsou et al., 2011 |
| STC-1 | paracrine factor | Mirotsou et al., 2011 |
| SDF-1 | paracrine factor | Mirotsou et al., 2011 |
| IGF-1 | paracrine factor | Mirotsou et al., 2011 |
| FGF2 | paracrine factor | Mirotsou et al., 2011 |
| TB4 | paracrine factor | Mirotsou et al., 2011 |
| TNF-A | paracrine factor | Mirotsou et al., 2011 |
| Ang-1 | paracrine factor | Mirotsou et al., 2011 |
| Ang-2 | paracrine factor | Mirotsou et al., 2011 |
| PIGF | paracrine factor | Mirotsou et al., 2011 |
| MCP-1 | paracrine factor | Mirotsou et al., 2011 |
| PDGF-BB | paracrine factor | Mirotsou et al., 2011 |
| MMP-2 | paracrine factor | Mirotsou et al., 2011 |
| MMP-9 | paracrine factor | Mirotsou et al., 2011 |
| TSP1 | paracrine factor | Mirotsou et al., 2011 |
| TIMP-1 | paracrine factor | Mirotsou et al., 2011 |
| TIMP-2 | paracrine factor | Mirotsou et al., 2011 |
| TIMP-9 | paracrine factor | Mirotsou et al., 2011 |
| NGF | paracrine factor | Mirotsou et al., 2011 |
| ERBB2 | paracrine factor | Mirotsou et al., 2011 |
| IDO | paracrine factor | Liang et al., 2014 |
| HMOX1 | paracrine factor | Liang et al., 2014 |
| NO | paracrine factor | Liang et al., 2014 |
| HLA-G | paracrine factor | Liang et al., 2014 |
| PGE2 | paracrine factor | Liang et al., 2014 |
| BCL2 | paracrine factor | Liang et al., 2014 |
| BIRC5 | paracrine factor | Liang et al., 2014 |
| CSF2 | paracrine factor | Liang et al., 2014 |
| GDNF | paracrine factor | Liang et al., 2014 |
| LIF | paracrine factor | Liang et al., 2014 |
